# Probing ligand binding of endothiapepsin by ‘temperature-resolved’ macromolecular crystallography

**DOI:** 10.1107/S205979832200612X

**Published:** 2022-07-27

**Authors:** Chia-Ying Huang, Sylvain Aumonier, Sylvain Engilberge, Deniz Eris, Kate Mary Louise Smith, Filip Leonarski, Justyna Aleksandra Wojdyla, John H. Beale, Dominik Buntschu, Anuschka Pauluhn, May Elizabeth Sharpe, Alexander Metz, Vincent Olieric, Meitian Wang

**Affiliations:** a Swiss Light Source, Paul Scherrer Institut, Forschungsstrasse 111, Villigen-PSI, 5232 Villigen, Switzerland; b Idorsia Pharmaceuticals Ltd, Hegenheimermattweg 91, 4123 Allschwil, Switzerland

**Keywords:** conformational heterogeneity, protein plasticity, fragment binding, endothiapepsin, room temperature macromolecular crystallography

## Abstract

Continuous developments in cryogenic X-ray crystallography have provided most of our knowledge of 3D protein structures, which has recently been further augmented by revolutionary advances in cryoEM. However, a single structural conformation identified at cryogenic temperatures may introduce a fictitious structure as a result of cryogenic cooling artefacts, limiting the overview of inherent protein physiological dynamics, which play a critical role in the biological functions of proteins. Here, a room-temperature X-ray crystallo­graphic method using temperature as a trigger to record movie-like structural snapshots has been developed. The method has been used to show how TL00150, a 175.15 Da fragment, undergoes binding-mode changes in endothiapepsin. A surprising fragment-binding discrepancy was observed between the cryo-cooled and physiological temperature structures, and multiple binding poses and their interplay with DMSO were captured. The observations here open up new promising prospects for structure determination and interpretation at physiological temperatures with implications for structure-based drug discovery.

## Abstract 2

A room-temperature X-ray crystallographic method using temperature as a trigger to record movie-like structural snapshots has been developed and applied to study ligand binding and protein plasticity.

## 1. Introduction

Macromolecular X-ray (MX) crystallography at 100 K is the conventional approach for determining atomic protein crystal structures. Cryo-cooling the protein crystal reduces the propensity for radiation damage and enhances crystal lifetimes (Garman & Schneider, 1997 ▸; Garman, 2003 ▸). The success of cryo-MX in terms of beamtime efficiency gains cannot be overstated. Fully automated, high-throughput data collection from many hundreds of crystals per synchrotron shift is now routine and is the *modus operandi* of modern drug development (Maveyraud & Mourey, 2020 ▸).

The utility of these data is based on the assumption that protein crystal structures at 100 K are a fair representation of the protein in its physiological environment. However, evidence is mounting that structures determined at 100 K can suppress conformational heterogeneity, which could be an integral part of enzymatic reactions (Halle, 2004 ▸; Keedy *et al.*, 2015 ▸; Thomaston *et al.*, 2017 ▸; Broom *et al.*, 2020 ▸; Yabukarski *et al.*, 2020 ▸), and create artificial ligand-binding modes (Fraser *et al.*, 2011 ▸; Fischer *et al.*, 2015 ▸; Fischer, 2021 ▸). Room-temperature (RT) X-ray structures may represent more functionally relevant conformations that could be critical for pharma­ceutical drug discovery. Recently, multiple-temp­erature crystallography from 100 K to the glass transition at 200 K and in the physiological range 270–330 K has been used to successfully track protein conformation changes and ligand binding (Gómez & Freire, 1995 ▸; Weik & Colletier, 2010 ▸; Keedy *et al.*, 2015 ▸, 2018 ▸; Doukov *et al.*, 2020 ▸).

A key technique using high-throughput crystallographic methods is X-ray fragment-based drug discovery (xFBDD; Collins *et al.*, 2017 ▸). The protocol for successful xFBDD relies upon the rapid testing of thousands of weakly binding fragments to probe the chemical space of a prospective binding pocket (Congreve *et al.*, 2003 ▸) and potential allosteric sites. The necessity to screen thousands of crystals rapidly has effectively limited fragment-screening methods to cryo-temperatures. Until recently, only cryo-methods were suited to the collection of large volumes of data from many thousands of protein crystals in a timely and efficient manner. However, xFBDD at RT could help the drug-development process by bridging the gap between cryo-MX and other screening techniques all performed at or above RT.

Fortunately, developments in software, detector speed and sensitivity, and beam flux, as well as RT devices and mounts, have made the application of routine MX at RT a more credible reality (Bowler *et al.*, 2015 ▸; Leonarski *et al.*, 2018 ▸; Doukov *et al.*, 2020 ▸; Fischer, 2021 ▸). Care still needs to be taken when selecting the best parameters to avoid radiation damage; however, several recent papers address such concerns, showing that if one remains conservative in the delivered X-ray dose and accepts a slightly lower resolution, radiation damage is not a limiting factor at RT in terms of structure interpretation. A guiding limit of 380 kGy has been proposed (de la Mora *et al.*, 2020 ▸). More importantly, studies have suggested that specific and global radiation damage are less decoupled at room temperature than at cryogenic temperatures, where specific damage is found to occur more quickly (Gotthard *et al.*, 2019 ▸), which makes it possible to measure protein conformational heterogeneity reliably (Russi *et al.*, 2017 ▸; Yabukarski *et al.*, 2022 ▸).

However, moving to purely RT methods is also a source of concern. As pointed out by Fischer *et al.* (2015 ▸), the propensity for binding ligands to occupy sites in crystal structures could be significantly increased by collecting data at 100 K (Fischer *et al.*, 2015 ▸). Therefore, the move to data collection at 293 K may not only give rise to more variability in ligand orientation or binding-site plasticity and greater dynamics of the ligand-bound state, but may also reduce the occupancy of sites. These factors make the practice of high-throughput xFBDD at RT more challenging as more care, and ultimately time, needs to be given to the structural interpretation and study of individ­ual fragments.

In this study, we report an optimized RT crystallographic approach that allows the monitoring of the temperature dependence of ligand occupancy at equilibrium around RT, and applied the method to a detailed analysis of the binding of a single fragment to a target protein. In this study, we used the aspartic protease endothiapepsin (EP), which serves as a model system for other aspartic proteases that play critical roles in several important diseases such as hypertension (renin), Alzheimer’s disease (β-secretase), malaria (plasmepsin IX/X) and HIV (HIV protease) (Cooper, 2002 ▸). EP has frequently been used to develop principles and fragment screening. Many fragment-based and inhibitor studies have found diverse ligand-binding modes in EP and, of these, ligand binding close to the catalytic centre of EP has been extensively studied (Köster *et al.*, 2011 ▸; Huschmann *et al.*, 2016 ▸; Schiebel *et al.*, 2016 ▸; Radeva *et al.*, 2016 ▸; Wollenhaupt *et al.*, 2020 ▸; Metz *et al.*, 2021 ▸). We investigated the mode and dynamics of the binding of fragment TL00150 to the active site of EP. Thanks to the availability of large and well diffracting EP crystals, a fast X-ray detector (Casanas *et al.*, 2016 ▸) and an optimized RT sample setup with a humidity-controlled chamber, we were able to capture a series of structural snapshots of EP-TL00150, and allowed the investigatation of the gradual evolution of its binding modes during temperature changes using helical-like data collection in a movie-like manner. Our work also revealed the interactive role of DMSO in TL00150 binding dynamics.

## 2. Materials and methods

### 2.1. Protein crystallization and fragment soaking

Endothiapepsin was purchased as Suparen 600 (Product No. 114604-516) from Prochem AG and was crystallized based on previously described work (Jenkins *et al.*, 1975 ▸; Köster *et al.*, 2011 ▸). In brief, the protein buffer was first exchanged into 100 m*M* sodium acetate pH 4.6 and concentrated to a final concentration of 10 mg ml^−1^ using a Merck Millipore Amicon ultracentrifugal filter unit (10 kDa cutoff). Crystallization trials were performed using a Mosquito robot (SPT Labtech) with MRC3 plates. Protein and crystallization solution were mixed in a 1:1 ratio to a final volume of 900 nl, and 20 nl microseeds (1:1000 dilution) were added to the drop before sealing. The crystallization solution consisted of 100 m*M* ammonium acetate, 100 m*M* sodium acetate pH 4.6, 26–30%(*v*/*v*) PEG 4000. The plates were stored at 20°C in an imager system (ROCK IMAGER 1000, Formulatrix). The crystals appeared in 24 h and grew to full size in 30–36 h. The rectangular-shaped crystals measured 600–800 × 80 × 40 µm in size.

The fragment TL00150 [4-(trifluoromethyl)benzylamine; catalogue No. 263508; Supplementary Table S1] was purchased from Sigma–Aldrich. Fragment soaking was performed using an Echo550 (Labcyte) at 20°C with targeted DMSO concentrations of 5%(*v*/*v*), 8%(*v*/*v*), 10%(*v*/*v*), 15%(*v*/*v*), 20%(*v*/*v*) and 40%(*v*/*v*), which are used throughout the paper. The final TL00150 concentration in the crystal drop was between 40 and 63 m*M*, with DMSO concentrations of 4.4%(*v*/*v*), 7.0%(*v*/*v*), 9.4%(*v*/*v*), 14.0%(*v*/*v*), 19.3%(*v*/*v*) and 39.1%(*v*/*v*). The crystals were soaked for 6–9 h and were then harvested for cryo, RT or multi-temperature X-ray experiments.

### 2.2. Sample preparation and crystal harvesting

**Figure 1:**
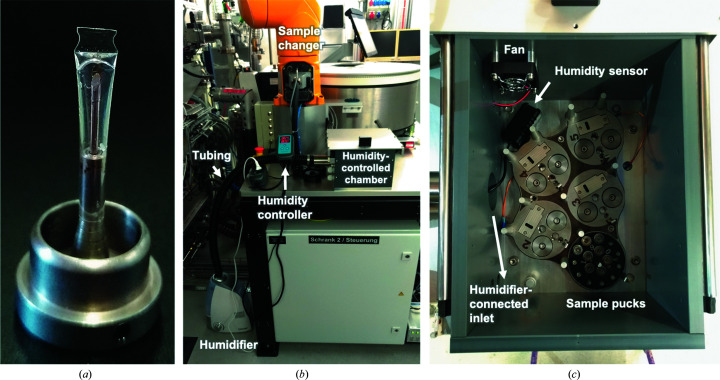
Sample setup and humidity-controlled chamber. (*a*) Crystal-harvesting loop on a magnetic base with the protection of a plastic capillary. (*b*) Overview of the humidity-controlled device at PXII-X10SA. (*c*) The internal details of the humidity-controlled RT box.

The crystals for the RT data collections were mounted using MicroLoops E^TM^ (SKU M8-L18SP-50V), reusable B5 gonio­meter bases (SKU GB-B5-R-20) and MicroRT capillaries (SKU RT-T1) (Fig. 1 ▸ *a*), all from MiTeGen, Ithaca, New York, USA. The MicroRT capillaries were trimmed to 17 mm so that the total length of the assembly (loop, base and capillary) remained compatible with the TELL sample exchanger (Martiel *et al.*, 2020 ▸) at the PXII-X10SA beamline, the gripper cavity of which can accommodate a maximum sample length of 31.2 mm.

**Figure 2:**
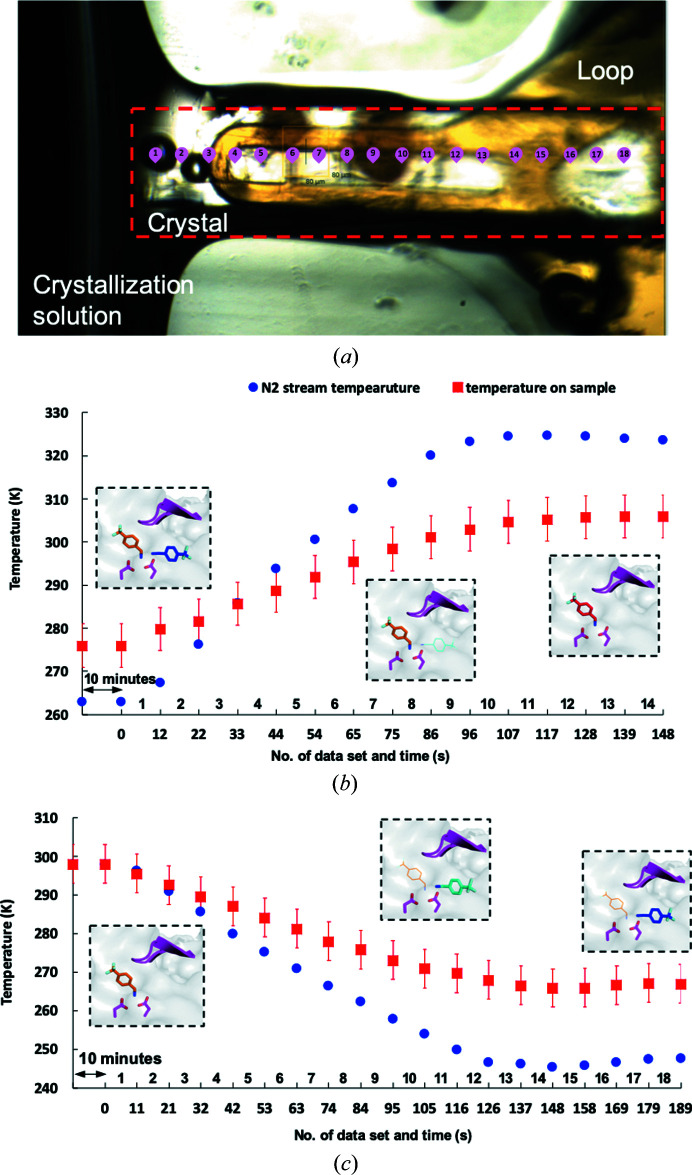
Helical-like data collection and timestamp for data sets with the corresponding temperature. (*a*) An enlarged view of a crystal harvested in a loop with the crystal tip slightly touching the reservoir solution. A rectangular red dashed line outlines the crystal and the bookmarked positions are illustrated in pink with the data-set numbers. (*b*, *c*) The timestamp of the data sets and the corresponding temperatures for the temperature ramp-up at 15%(*v*/*v*) DMSO and ramp-down at 5%(*v*/*v*) DMSO experiments, respectively. The blue circles and red squares represent the N_2_ stream temperature and the estimated actual temperature at the sample, respectively. The numbers above and below the *x* axis indicate the number of the data set and the time, respectively. The 10 min indicated in the figures is the equilibration time for the experiment temperature before data collection. The arrow lines in the figures are indicative and are not drawn on the scale of the real-time line.

To maintain crystal hydration, we added 1.5–2 µl of the final crystallization solution, with comparable concentrations of TL00150 and DMSO as in the soaked crystal, to the top of the capillary. The crystals were harvested so that their longer face is oriented parallel to the mounting pin, facilitating data collection. The loop with the crystal was then quickly inserted into the trimmed MicroRT capillaries with the crystal tip slightly touching the reservoir solution (Fig. 2 ▸ *a*). The assembled samples were then stored in a UniPuck. Once fully loaded (approximately 15 min), the puck was placed into the humidity-controlled chamber installed at the PXII-X10SA beamline with the humidity set to 80% for longer term storage (Figs. 1 ▸ *b* and 1 ▸ *c*).

For reference, crystals were also harvested and snap-cooled in liquid nitrogen using conventional cryo-methods. The loops were stored in UniPucks in the beamline Dewar for the experiment.

### 2.3. Data collection

X-ray diffraction data were collected on beamline PXII-X10SA at the Swiss Light Source (SLS), Villigen-PSI, Switzerland. The sample temperature was controlled by a cryostream (Cryostream 800, Oxford) with a nozzle-to-sample distance of 5 mm for the cryogenic experiments (100 K). A slightly longer distance of 8 mm was used for the RT (298 K) and multi-temperature experiments (263–323 and 298–248 K) to ensure sufficient clearance from the sample-mounting capillary. It is important to note that the capillary sample enclosure makes the actual crystal temperature differ from the set N_2_ stream temperature. The actual sample temperatures were estimated using an infrared camera (the integrated FLIR thermal imaging camera on a Cat S61 telephone). The measurement point was at the sample inside the capillary and the corresponding temperatures are plotted in Figs. 2 ▸(*b*) and 2 ▸(*c*) for both temperature ramp-up and ramp-down scenarios. All samples were mounted using the TELL sample changer (Martiel *et al.*, 2020 ▸).

#### 2.3.1. ‘Room temperature’ (RT) measurements

The RT X-ray diffraction data collection was designed to maintain a tolerable X-ray dose. A fast rotation (40 deg s^−1^) was used to mitigate radiation damage at RT by reducing the total data-collection time. The X-ray wavelength, beam transmission, flux and beam size were 1 Å (12.398 keV), 20%, ∼4 × 10^11^ photons s^−1^ and 80 × 80 µm, respectively. The EIGER2 16M detector was operated at a 100 Hz frame rate with a sample-to-detector distance of 135 mm. The accumulated average diffraction-weighted X-ray dose was estimated by *RADDOSE*-3*D* (Zeldin *et al.*, 2013 ▸) as about 84 kGy for a 360° data set, which is below the radiation-damage safety margin for RT MX (Southworth-Davies *et al.*, 2007 ▸; Owen *et al.*, 2012 ▸; Warkentin *et al.*, 2012 ▸, 2017 ▸; Roedig *et al.*, 2016 ▸). Note that such a dose could heat up the crystals slightly (Warren *et al.*, 2019 ▸). As a control, we also collected a second data set with the same dose from the same crystal position. The data-processing and refinement statistics are comparable between the two measurements (data not shown), indicating that the applied dose is safe for this study and potential crystal dehydration effects are minimal.

#### 2.3.2. Cryo-temperature measurements

For the cryo data sets, the crystals were stored in liquid nitrogen and measured at 100 K. The X-ray wavelength, beam transmission, collection speed, flux and beam size were 1 Å (12.398 keV), 100%, 50 deg s^−1^, ∼2 × 10^12^ photons s^−1^ and 80 × 80 µm, respectively. The detector and sample-to-detector distance were the same as for the RT setup. The accumulated average diffraction-weighted X-ray dose was estimated by *RADDOSE*-3*D* (Zeldin *et al.*, 2013 ▸) as about 459 kGy for a 360° data set.

#### 2.3.3. Temperature ‘ramp-up’ and ‘ramp-down’ measurements

For the temperature ramp-up and ramp-down experiments, the sample was first equilibrated with the N_2_ stream at either 263 K (ramp-up) or 298 K (ramp-down) for 10 min. Whilst the sample was equilibrating, multiple data-collection points (bookmarks; Wojdyla *et al.*, 2018 ▸) were made along the long axis of the cuboid crystal (Fig. 2 ▸ *a*). The data collection and the N_2_-stream-controlled temperature change were then launched simultaneously such that diffraction data were continuously collected during the sample-temperature change. A 360° data set from each bookmark was collected in 9 s and the translation time between two bookmark positions was about 1.5 s (Figs. 2 ▸ *b* and 2 ▸ *c*). A control experiment was performed with a similar crystal without changing the temperature, and there was no difference in the fragment position among all of the data sets (data not shown).

For the temperature ramp-up experiment, the N_2_ stream temperature was increased from 263 to 323 K (Fig. 2 ▸ *b*). 14 bookmarks were made along a 600 × 80 × 40 µm EP crystal with a total data-collection time of 148 s. The N_2_ stream temperature started at 263 K at time point 0 and reached 323 K after 96 s. The temperature was then maintained at 323 K until the end of data collection at 148 s. The actual sample temperatures were estimated to be 276, 303 and 306 K at time points 0, 96 and 148 s, respectively (Fig. 2 ▸ *b*).

For the temperature ramp-down experiment, the N_2_ stream temperature was decreased from 298 to 248 K (Fig. 2 ▸ *c*). 18 bookmarks were made along the 800 × 80 × 40 µm EP crystal for a total data-collection time of 189 s. The N_2_ stream temperature started at 298 K at time 0, reached 248 K after 126 s and continued at 248 K until the end of data collection at 189 s. The actual sample temperatures were found to be 298, 268 and 267 K at times of 0, 126 and 189 s, respectively (Fig. 2 ▸ *c*).

### 2.4. Data processing

The single data sets from Sections 2.3.1 and 2.3.2 and a data set at each bookmark position from Section 2.3.3 were processed separately with the same input processing parameters using *XDS* and scaled using *XSCALE* (Kabsch, 2010 ▸). All data sets were processed to resolutions of 1.8 Å for RT and multi-temperature experiments and 1.4 Å for cryogenic temperature measurements in space group *P*2_1_.

### 2.5. Structure determination, refinement and analysis

All structures were phased by molecular replacement using *Phaser* (McCoy *et al.*, 2007 ▸) with PDB entry 6rsv (F. Magari, A. Heine, M. Konstantinidou, F. Sutanto, J. Haupenthal, R. V. Jumde, M. Y. Unver, C. J. Camacho, A. K. H. Hirsch, A. Doemling & G. Klebe, unpublished work) as the search model. The structures were initially refined without any ligand using *BUSTER* (Bricogne *et al.*, 2017 ▸). This generated a difference map for locating the ligand. Subsequently, refinement of the EP–TL00150 complex was performed using three cycles of *phenix.refine* (Afonine *et al.*, 2012 ▸), refining the coordinates, restrained ADPs and occupancies. This was then followed by an additional five cycles of occupancy-only refinement. The coordination of TL00150 was generated with *phenix.elbow* (Moriarty *et al.*, 2009 ▸) using NCc1ccc(C(F)(F)F)cc1 as the SMILES file input (Supplementary Table S1). Manual building of the protein model and ligand was carried out using *Coot* (Emsley *et al.*, 2010 ▸). Data-collection, processing and refinement statistics are reported in Supplementary Tables S2–S5. Structures and associated structure-factor amplitudes have been deposited in the Protein Data Bank (PDB; Berman *et al.*, 2000 ▸) with accession codes 7qm3, 7qm4, 7qm5, 7qm6, 7qm7, 7qm8 (Supplementary Table S2), 7qly, 7qlz, 7qm0, 7qm1, 7qlu, 7qlv, 7qlw, 7qlx, 7qlt (Supplementary Table S3), 7qmr, 7qms, 7qmt, 7qmu, 7qmv, 7qmw, 7qmx, 7qmy, 7qmz, 7qn0, 7qn1, 7qn2, 7qn3, 7qn4 (Supplementary Table S4), 7qm9, 7qma, 7qmb, 7qmc, 7qmd, 7qme, 7qmf, 7qmg, 7qmh, 7qmi, 7qmj, 7qmk, 7qml, 7qmm, 7qmn, 7qmo, 7qmp and 7qmq (Supplementary Table S5). Figures were generated with *PyMOL* (version 1.8.4.0; Schrödinger).

Within *Konstanz Information Miner* (*KNIME*) version 4.3.2 (Berthold *et al.*, 2008 ▸), all EP structures from this study and the PDB (Supplementary Table S6) were prepared with the *Schrödinger Preparation Wizard* (Schrödinger; default setting without energy minimization) and superimposed onto a common apo reference structure (PDB entry 4y5l; J. Schiebel, A. Heine & G. Klebe, unpublished work). Overlapping ligand and DMSO binding poses were identified by *Schrödinger Shape Screening* (default setting with typed atoms), keeping all poses in the previously aligned protein structures unchanged.

## 3. Results

### 3.1. Experimental setup for RT MX

Prior to data collection we stored samples in a humidity-controlled chamber that can maintain a constant relative humidity of 80% around the sample (Fig. 1 ▸). The device is equipped with a humidifier, a chamber, a humidity sensor, a fan and five UniPuck positions. The humidifier and humidity-controlled chamber were connected by a tube. We set the relative humidity value to 80%, which had been found to satisfactorily preserve test samples in a sandwich-chip setup for up to seven days (Supplementary Fig. S1; Karpik *et al.*, 2020 ▸). A fan on the wall of the chamber was found to be necessary to more accurately maintain a desired humidity level. The crystals were harvested in capillary-enclosed loops with the crystal tip slightly touching the reservoir solution (Fig. 2 ▸ *a*), which has been found to preserve the crystal for a longer period of time during data collection. Using this setup, a total of 80 samples could be stored and screened with the aid of the TELL sample changer.

### 3.2. Structure of the EP–TL00150 complex in different DMSO concentrations at 298 K and 100 K

TL00150 was selected from a previous fast fragment compound-screening (FFCS) study (work to be published). Since it is known that DMSO can be a competitive binder in fragment-based screening (Navratilova *et al.*, 2016 ▸), we first measured the EP–TL00150 complex at both RT (298 K) and at 100 K at different DMSO concentrations from 5%(*v*/*v*) to 40%(*v*/*v*). A reference control experiment was then performed using EP crystals with only DMSO at both 298 and 100 K to avoid the crystallization solution dilution effect from soaking.

**Figure 3:**
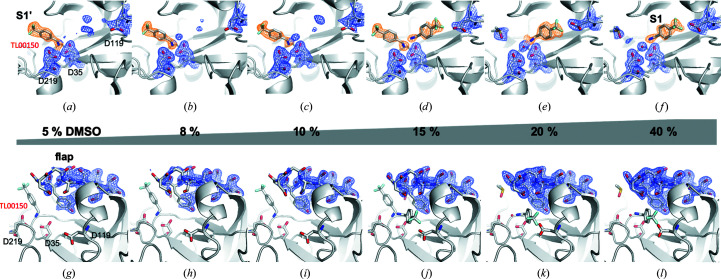
Comparison of 2*F*_o_ − *F*_c_ electron-density maps at 298 K around (*a*)–(*f*) the active site and (*g*)–(*l*) the flap domain (residues Ser76–Ser86) of EP–TL00150 structures as a function of DMSO concentration. The catalytic dyad, Asp35 and Asp219, and also Asp119, TL00150 and DMSO, are shown as stick representations. The S1 and S1′ positions of the EP active-site pocket and flap domain are shown in (*f*), (*a*) and (*g*), respectively. The 2*F*_o_ − *F*_c_ electron-density maps contoured at 1σ are shown in orange for TL00150 and in blue for the rest of the residues and DMSO. The concentration trend of DMSO is indicated by a grey triangular bar. Lys110–Ser120 are omitted from the figures for clarity.

**Figure 4:**
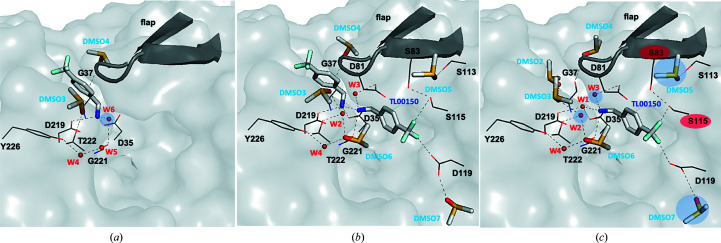
Molecular interactions of TL00150 and EP. Molecular interactions of EP–TL00150 in (*a*) S1′, (*b*) both S1′ and S1 and (*c*) S1 of EP. All relevant hydrogen bonds are depicted as dashed lines. The flap domain, the S1 and S1′ positions of the EP active-site pocket and residues involved in the hydrogen-bonding network are shown. The DMSO and water molecules with a blue shadow are not observed in some RT structures. The residues with a red shadow show an alternative conformation in 100 K structures.

Interestingly, at 298 K TL00150 was found to have two binding positions in the active site of EP depending on the DMSO concentration (Fig. 3 ▸ and Supplementary Table S2). When the DMSO concentration is below 15%(*v*/*v*), TL00150 binds in the S1′ position of the EP active-site pocket (Fig. 4 ▸ *a*). The binding of TL00150 at this S1′ position is mainly stabilized via electrostatic interactions between its amino moiety, which replaces the catalytic water (W1), and the catalytic dyad (Asp35 and Asp219). Another binding position was found in the EP–TL00150 structures with 20%(*v*/*v*) and 40%(*v*/*v*) DMSO. Here, TL00150 is bound to the S1 active-site pocket of EP, where the amino moiety of TL00150 forms a hydrogen-bonding network with the catalytic dyad (Asp35 and Asp219), the main-chain O atoms of Gly221 and Thr222, DMSO and water molecules, and the trifluoromethyl moiety makes multiple contacts with Ser115 and Asp119 of EP (Fig. 4 ▸ *c*). In addition, TL00150 also forms hydrogen bonds to Gly80/Asp81 of the flap domain (a flexible loop of residues 76–86 covering the active site) via a water molecule (W3), which is only seen in the 15–40%(*v*/*v*) DMSO EP–TL00150 structures (Fig. 4 ▸ *c*).

**Figure 5:**
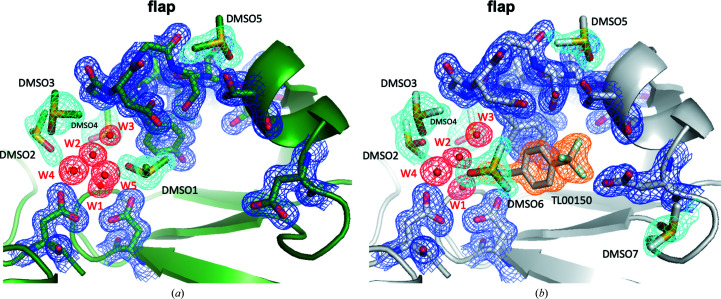
Comparison of EP and EP–TL00150 structures at 100 K. (*a*) EP structure with 10%(*v*/*v*) DMSO. (*b*) TL00150-soaked EP structure with 10%(*v*/*v*) DMSO. The r.m.s.d. (root-mean-square deviation) of EP with 10%(*v*/*v*) DMSO to TL00150-soaked EP with 10%(*v*/*v*) DMSO is 0.178 Å (residues 1–330). EP with 10%(*v*/*v*) DMSO and TL00150-soaked EP with 10%(*v*/*v*) DMSO are coloured green and grey, respectively. The flap domain, catalytic dyad (Asp35 and Asp219), Asp119, DMSO, water and TL00150 are depicted. The 2*F*_o_ − *F*_c_ electron-density maps contoured at 1σ are shown in blue for the flap domain, Asp35, Asp119 and Asp219, in cyan for DMSO molecules, in red for water and in orange for TL00150.

Both the S1 and S1′ binding positions have been identified in previous fragment-based screenings (Köster *et al.*, 2011 ▸; Huschmann *et al.*, 2016 ▸) of EP. The two binding positions are not mutually exclusive. We observed in the 15%(*v*/*v*) DMSO structure that TL00150 can partially occupy both the S1 and S1′ positions of the EP active-site pocket, in which the S1 TL00150 interacts with the S1′ TL00150 through its amino moiety in a hydrogen-bonding network with EP (Fig. 4 ▸ *b*). The flap domain of EP also shows flexible behaviour at 298 K. The flap-domain flexibility increases in the EP–TL00150 structures at lower DMSO concentrations [5–15%(*v*/*v*)], as indicated by the broken 2*F*_o_ − *F*_c_ map (Figs. 3 ▸ *g*–3 ▸ *j*). At higher concentrations of DMSO [20%(*v*/*v*) and 40%(*v*/*v*)] the flap domain is stabilized and shows better defined 2*F*_o_ − *F*_c_ density (Figs. 3 ▸ *k* and 3 ▸ *l*). In contrast, we found that TL00150 binds in a conserved way at the S1 position and that the flap-domain flexibility is much reduced in all EP–TL00150 structures at 100 K over the whole range of DMSO concentrations, indicating that the cryo-cooling process has suppressed these alternative conformations (Fig. 5 ▸).

To understand the importance of the role of DMSO in TL00150 binding, we analysed the DMSO positions in detail in structures of EP apo, EP with DMSO only and EP–TL00150 with DMSO determined at both 100 and 298 K (Supplementary Table S3). The DMSO molecules are assigned based on high-resolution electron-density maps and hydrogen-bonding networks (Fig. 5 ▸). The EP apo structures are both similar, with the flap domain in an ‘open’ conformation in the 100 and 298 K structures (Supplementary Figs. S2, S3*a* and S3*e* ). In the 100 K EP structures with 5–20%(*v*/*v*) DMSO but no fragment, five DMSO molecules were identified near or in the active-site pocket of the EP structure, and two of them (DMSO1 and DMSO2) are located in the vicinity of the S1 and S1′ TL00150 binding positions (Fig. 5 ▸ *a* and Supplementary Figs. S3*f*, S3*g* and S3*h* ). The flap domain is stabilized in a ‘close’ conformation by an extensive hydrogen-bonding network comprising four DMSO molecules (DMSO1–DMSO4) and four water molecules (W1–W4) (Fig. 5 ▸ *b* and Supplementary Fig. S3). Under the same conditions at 298 K the flap domain is largely flexible (modelled in both conformations in Supplementary Figs. S2 and S3*b*–S3*d*), indicating that the weaker water and DMSO binding at 298 K are not sufficient to lock the flap domain.

In all 100 K EP–TL00150 structures DMSO1 is replaced by TL00150 at the S1 position and a new DMSO6 binds by a hydrophobic interaction with the benzene ring of TL00150 (Fig. 5 ▸ *b*). DMSO6 and the amino moiety of TL00150 join the extensive hydrogen-bonding network between the catalytic residues and the tip of the flap domain. The refined occupancies of DMSO6 are 0.69, 0.72, 0.84, 0.82 and 0.92 in the structures with 5%(*v*/*v*), 10%(*v*/*v*), 15%(*v*/*v*), 20%(*v*/*v*) and 40%(*v*/*v*) DMSO, respectively. The flap domain is further stabilized by the trifluoromethyl moiety of TL00150 via interactions with Ser115. The side chain of Asp119 is re­directed by the trifluoromethyl moiety and creates a binding site for DMSO7. A similar structure with the flap domain in the closed conformation is conserved in the 298 K EP–TL00150 structures with 20%(*v*/*v*) and 40%(*v*/*v*) DMSO except that W2, W3 and some DMSO molecules are less ordered (Fig. 4 ▸ *c*). When the DMSO concentration is reduced in the 298 K EP–TL00150 structures, the extensive hydrogen-bonding network gradually deteriorates, the flap domain is released to the open position (Figs. 3 ▸ *g*, 3 ▸ *h*, 3 ▸ *i* and 3 ▸ *j*) and TL00150 moves out from the S1 to the S1′ binding position. These observations strongly support the observed binding modes being induced by both the TL00150 and the DMSO concentration.

### 3.3. Temperature dependence of TL00150 binding near room temperature

TL00150 shows two binding positions in EP–TL00150 at 298 K depending on the DMSO concentration, while it is only stabilized at the S1 position of the EP active-site pocket at 100 K. This provided the opportunity to use a temperature change as a trigger to record movies of the different binding modes. To demonstrate this, we performed two experiments with continuous temperature changes on samples to monitor the behaviour of TL00150. The initial temperature of the sample was controlled by the N_2_ stream at the beamline sample position. An infrared camera was then used to estimate the actual temperature of the sample in the stream (details are described in Section 2.3.3). Only the infrared measured temperatures are referred to in Sections 3.3.1 and 3.3.2.

#### 3.3.1. Temperature ramp-up experiment from 276 to 306 K

EP–TL00150 crystals with 15%(*v*/*v*) and 20%(*v*/*v*) DMSO were chosen for the temperature ramp-up experiment to determine whether TL00150 binds to the S1′ position as the temperature rises. 14 data sets were collected continuously in the course of the temperature-ramping experiment (Fig. 2 ▸ *b* and Supplementary Table S4).

**Figure 6:**
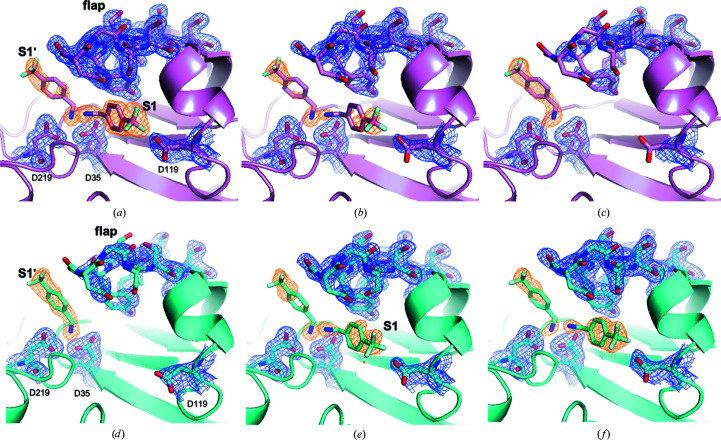
Comparison of the electron-density maps of TL00150 and the flap domain in ramp-up and ramp-down experiments. The electron density of TL00150 and surrounding EP residues is shown at (*a*) 276 K (ramp-up data set 1), (*b*) 303 K (ramp-up data set 9), (*c*) 306 K (ramp-up data set 14), (*d*) 298 K (ramp-down data set 1), (*e*) 268 K (ramp-down data set 12) and (*f*) 267 K (ramp-down data set 18). The 2*F*_o_ − *F*_c_ electron-density maps contoured at 1σ are shown in orange for TL00150 and in blue for the rest of the residues. The temperatures discussed here were measured from the sample using an infrared camera (details are described in Section 2.3).

The result showed that the TL00150 did not undergo any change in binding position during the temperature ramp-up from 276 to 306 K and remained in the S1 position in the EP–TL00150 complex with 20%(*v*/*v*) DMSO (data not shown). This could be explained by the flap domain being strongly stabilized by both TL00150 and DMSO, hence locking TL00150 in the EP active-site pocket (Fig. 4 ▸ *c*). When a reduced DMSO concentration of 15%(*v*/*v*) was used for the same experiment, the flap domain became more disordered (Fig. 3 ▸ *j*). At 276 K, TL00150 occupied both the S1 and S1′ positions of the EP active-site pockets. The S1 TL00150 forms a stable hydrogen-bonding network via its amino moiety to S1′ TL00150 (Fig. 6 ▸ *a*). In addition, the carboxyl group of Asp119 is orientated towards the S1 TL00150 and has well defined electron density (Fig. 6 ▸ *a*). The electron density of the S1′ TL00150 is clear but relatively weak, suggesting a possible mixed occupancy of TL00150 and DMSO2/catalytic water. When the temperature was ramped up, the density of the S1 TL00150 started to disappear (Fig. 6 ▸ *b*) and eventually vanished in the last two data sets (Fig. 6 ▸ *c*). At the same time, the carboxyl group of Asp119 and the flap domain became gradually disordered (Figs. 6 ▸ *a*–6 ▸ *c*). With the S1′ position fully occupied by TL00150, the end state of this series largely resembles the EP–TL00150 structure with 5%(*v*/*v*) DMSO at 298 K (Figs. 3 ▸ *a* and 3 ▸ *g*).

The refined occupancies of TL00150 in the S1 and S1′ positions reflect the binding-mode evolution (Supplementary Fig. S4*a* ). As expected, the average *B* factor for both TL00150 positions increased slightly when the temperature was ramped up (Supplementary Fig. S4*b* ). The TL00150 occupancies remain relatively high, even when the broken electron density suggested that TL00150 had almost left the binding site. We attribute this effect to the fact that the binding sites are occupied most of the time by either TL00150 or DMSO/water. The entire process is also presented as a movie in which 14 successive structures capture a sequence of events where TL00150 first binds at both the S1 and S1′ positions (low temperature) to progressively being bound only to the S1′ position (high temperature) of the EP active-site pocket (Supplementary Movie S1).

#### 3.3.2. Temperature ramp-down experiment from 298 to 267 K

In Section 3.3.1 we showed that TL00150 had a tendency to bind at the S1′ position when the temperature was close to RT and the DMSO concentration was low. To observe this process in the reverse direction, we conducted a temperature ramp-down experiment with EP–TL00150 in 5%(*v*/*v*) DMSO where TL00150 was initially only bound in the S1′ position of the EP active-site pocket (Figs. 3 ▸ *a* and 3 ▸ *g*).

18 data sets (Supplementary Table S5) were collected during the cooling from 298 to 267 K, and they recorded the expected reverse process as shown in Figs. 6 ▸(*d*)–6 ▸(*f*) and Supplementary Movie S2. Briefly, at 298 K TL00150 initially only occupied the S1′ position of the EP active-site pocket (Fig. 6 ▸ *d*). Increasing occupancy of TL00150 at the S1 position was observed as the temperature decreased (Figs. 6 ▸ *e* and 6 ▸ *f*), as also reflected in the analysis of the gradually decreasing *B* factor (Supplementary Figs. S4*c* and S4*d* ). The final 267 K structure is similar to the 100 K structure.

## 4. Discussion

In this study, we extended multi-temperature X-ray crystallography to temperature-resolved crystallography. We adapted our cryogenic sample-changer system (TELL) to mount RT samples by adding a compact RT sample-storage system. With the ease of RT sample handling, we performed temperature-ramping X-ray crystallography on EP crystals by combining the fast frame rate of the EIGER2 16M detector and the cryojet system installed on PXII-X10SA at the SLS.

A series of EP structures with the bound fragment TL00150 were recorded to assess the occupancy of the fragment and the evolution of its location throughout the temperature range. The movies show multiple fragment-binding states, highlighting the importance of the flexible loops (flap domain) of EP and offering help in understanding the catalytic mechanism at physiological temperature. In comparison the 100 K structures only show a single stabilized protein state, which could have been introduced by the cryo-cooling procedure. In addition, we found different binding modes for TL00150 between the S1 and S1′ positions in the EP structure that varied as a function of temperature and DMSO concentration. Ramping up the temperature during the X-ray crystallography experiment induces a change of occupancy in the S1 and S1′ positions, and ramping it down reverses it. This suggests the possibility of monitoring ligand-binding changes in response to temperature change within a crystal using ‘temperature-resolved’ X-ray crystallography.

Interestingly, in the temperature ramp-up/down experiment of the EP–TL00150 complex, the binding interaction network of TL00150 gradually changes as the temperature increases or decreases, leading to multiple binding states. TL00150 binds at the S1′ position mainly at RT or higher, as well as at low DMSO concentrations. The interaction is made through its amino moiety, which mimics the natural substrate (peptides) and replaces the catalytic water molecule. To better understand the binding mode of TL00150 in EP, we aligned and compared 256 cryogenic EP ligand (fragment and compounds) structures from the Protein Data Bank (Supplementary Fig. S5). TL00150 binds to the active-site cleft of EP, in which 240 ligands were seen to bind. Several of the fragment structures had a similar binding mode to TL00150, with PDB entry 4y4w having the most similar binding interaction (Supplementary Fig. S6; Radeva *et al.*, 2016 ▸). However, at cryogenic temperatures protein plasticity can be limited due in part to the higher energy barrier that needs to be crossed to allow fragment binding when compared with room temperature. In the latter the protein has a more flexible behaviour, as often shown by the presence of multiple conformers of residue side chains, and therefore the binding energy barrier required for fragment binding or different binding modes is lowered (Bradford *et al.*, 2021 ▸). Our temperature-ramping MX setup could be a useful tool to probe serial binding modes of protein and ligand at atomic resolution around RT instead of a single state from a cryogenic structure. We expect that such temperature-snapshot structures will play an increasingly important role in the future development of MX at a time when most static protein structures can be predicted by *AlphaFold*2 (Jumper *et al.*, 2021 ▸) and *RoseTTaFold* (Baek *et al.*, 2021 ▸).

In xFBDD applications, as fragments typically bind weakly to target proteins (typical *K*_d_ values of 10^−2^ to 10^−4^ *M*), high concentrations of fragments must be used in order to achieve an appreciable saturation of binding. This often requires the use of co-solvents to increase solubility. DMSO is usually used for this purpose. Before beginning a fragment-screening experiment, it is advisable to ascertain the impact of DMSO concentration on the fragment-soaking efficiency and protein stability. DMSO has been observed to compromise the detection of low binding-affinity fragments in biosensor fragment screening (Navratilova *et al.*, 2016 ▸) and can even block the binding of weaker fragments if it binds sufficiently tightly in the active site (Bedi *et al.*, 2020 ▸). In addition, very high DMSO concentrations could shift the stability of the proton­ation states and interactions of chargeable groups involved in protein–ligand interactions. Indeed, we observed a combined/correlation effect of DMSO and TL00150 on the flap-domain conformation and binding mode. This suggests that it is important to consider not only the effect of DMSO on the diffraction quality of the crystals but also the potential implications for downstream fragment elongation/combination.

The experimental setup and the data-collection parameters need to be considered when using MX at RT. Issues such as crystal dehydration, heating from the beam and radiation damage may be the source of misleading results. The development of serial crystallography using either free-electron lasers or synchrotron sources has provided routes to help eliminate or mitigate radiation damage using novel sample-delivery methods such as injector and fixed targets (Diederichs & Wang, 2017 ▸; Stauch *et al.*, 2018 ▸; Pearson & Mehrabi, 2020 ▸). For single-crystal methods, humidity-controlled devices for crystal harvesting and X-ray data collection have been developed such as the free-mounting system (Kiefersauer *et al.*, 2000 ▸) or the more recent HC-lab (Sanchez-Weatherby *et al.*, 2009 ▸; Bowler *et al.*, 2015 ▸). The latter has found an interesting application in the development of a time-resolved oscillation crystallography method (Aumonier *et al.*, 2020 ▸). The harvesting and mounting of room-temperature crystals at the beamline can also be automated by combining the HC-lab with the CrystalDirect harvester (Felisaz *et al.*, 2019 ▸). Another effective way to keep the crystal hydrated is to use an inert oil (Paratone N) to cover the entire crystal and surrounding liquid, as recently demonstrated (Doukov *et al.*, 2020 ▸; Yabukarski *et al.*, 2020 ▸).

In this study, we combined classic capillary sample mounting (Skrzypczak-Jankun *et al.*, 1996 ▸) with a humidity-controlled chamber and a sample changer for semi-automated RT data collection. Here, a temperature ramp-up or ramp-down experiment on a single crystal can be measured within 3 min. This experiment has been made possible by crystallization optimization work leading to highly reproducible and conserved large crystals, allowing the collection of multiple-temperature data sets per crystal that are comparable to one another. In the case of smaller crystals, fewer temperature variations can be collected at once, and the experiment needs to be repeated and sliced in smaller temperature ranges. This reduces the productivity rate of the method and increases the number of crystals required. However, if the crystalline system has been optimized to obtain isomorphous and comparable crystals, the humidity-controlled chamber combined with automated mounting enables the experiment to be performed in an efficient and straightforward way even in such cases. In the case where one temperature point can only be collected using several crystals, the data-processing strategy will need to be adapted to enable the merging of compatible data sets, which can be identified by parameters such as ISa and CC_1/2_ (Diederichs, 2009 ▸; Karplus & Diederichs, 2012 ▸; Santoni *et al.*, 2017 ▸). One drawback of our alternative setup to the humidity controller at the goniometer position is that in the higher temperature range (above RT, *i.e.* 298 K) dehydration of the crystal can be observed within the capillary. It has been shown that crystal dehydration can influence side-chain conformational heterogeneity by affecting the unit cell and crystal contacts and therefore needs to be taken into consideration when performing room-temperature experiments (Atakisi *et al.*, 2018 ▸). To be consistent and to be able to compare data sets between multiple temperatures, we purposely harvested crystals of a similar size and set up the capillary on the loop in such a way that the crystal and the capillary reservoir remain in close contact, thereby avoiding dehydration within the experimental time frame (Fig. 2 ▸ *a*). The difference in *I*/σ(*I*) and resolution range between the cryogenic and RT data in the highest resolution shell can be attributed to the use of different doses. The data sets collected at 298 K and temperature ramp-up/down structures show no significant difference in data quality, as shown by a constant unit-cell volume and mosaicity and similar resolution (Supplementary Tables S4 and S5). Although a few data sets have slightly lower *I*/σ(*I*) and higher *R*_meas_, these have a minor and insignificant influence on structure refinement and electron-density generation. This crystal-mounting procedure requires good dexterity, adds a supplementary and time-consuming step in sample preparation and could damage fragile crystals. To overcome some of these shortcomings, our workflow can be readily combined with the oil-based crystal-mounting method to gain better control of the humidity and achieve more precise temperature control on the crystal.

In addition to temperature range, the ramping speed can also be adjusted to fit experimental purposes. The structure-sampling points can be tuned by matching the speed of temperature ramping and data collection. While the EIGER 16M detector was operated at 100 Hz in this work, faster EIGER detectors (Förster *et al.*, 2019 ▸) and newer-generation detectors such as JUNGFRAU (Leonarski *et al.*, 2018 ▸) can reach frame rates of 1 kHz or higher, which could be used to track faster structural changes. The simple method presented here allows the use of temperature as a trigger to probe binding-mode changes and infer dynamics. When coupled with chemical probes (Garbaccio & Parmee, 2016 ▸) or fragment-based approaches (Keedy *et al.*, 2018 ▸) to interrogate structure–function relationships, this method could provide an appealing avenue to improve our understanding of mechanisms. We hope that this work and its future adaptations could promote studies of protein structural changes using macromolecular crystallography since the experimental setup and procedure are easily adaptable to any MX beamline.

## Supplementary Material

PDB reference: endothiapepsin–TL00150 complex, 7qlt

PDB reference: 7qlu

PDB reference: 7qlv

PDB reference: 7qlw

PDB reference: 7qlx

PDB reference: 7qly

PDB reference: 7qlz

PDB reference: 7qm0

PDB reference: 7qm1

PDB reference: 7qm3

PDB reference: 7qm4

PDB reference: 7qm5

PDB reference: 7qm6

PDB reference: 7qm7

PDB reference: 7qm8

PDB reference: 7qm9

PDB reference: 7qma

PDB reference: 7qmb

PDB reference: 7qmc

PDB reference: 7qmd

PDB reference: 7qme

PDB reference: 7qmf

PDB reference: 7qmg

PDB reference: 7qmh

PDB reference: 7qmi

PDB reference: 7qmj

PDB reference: 7qmk

PDB reference: 7qml

PDB reference: 7qmm

PDB reference: 7qmn

PDB reference: 7qmo

PDB reference: 7qmp

PDB reference: 7qmq

PDB reference: 7qmr

PDB reference: 7qms

PDB reference: 7qmt

PDB reference: 7qmu

PDB reference: 7qmv

PDB reference: 7qmw

PDB reference: 7qmx

PDB reference: 7qmy

PDB reference: 7qmz

PDB reference: 7qn0

PDB reference: 7qn1

PDB reference: 7qn2

PDB reference: 7qn3

PDB reference: 7qn4

Supplementary Figures, Supplementary Tables and captions to Supplementary Movies. DOI: 10.1107/S205979832200612X/rs5003sup1.pdf

Supplementary Movie S1. DOI: 10.1107/S205979832200612X/rs5003sup2.mp4

Supplementary Movie S2. DOI: 10.1107/S205979832200612X/rs5003sup3.mp4
