# Supplementary material for: Probing ligand binding of endothiapepsin by ‘temperature-resolved’ macromolecular crystallography
Source: Acta Crystallogr D Struct Biol. 2022 Jul 27;78(Pt 8):964–74. doi: 10.1107/S205979832200612X (PMC9344481; doi:10.1107/S205979832200612X)

STRUCTURAL BIOLOGY

**Volume 78 (2022)**

**Supporting information for article:**

# Probing ligand binding of endothiapepsin by `temperature-resolved' macromolecular crystallography

**Chia-Ying Huang, Sylvain Aumonier, Sylvain Engilberge, Deniz Eris, Kate Mary Louise Smith, Filip Leonarski, Justyna Aleksandra Wojdyla, John H. Beale, Dominik Buntschu, Anuschka Pauluhn, May Elizabeth Sharpe, Alexander Metz, Vincent Olieric and Meitian Wang**

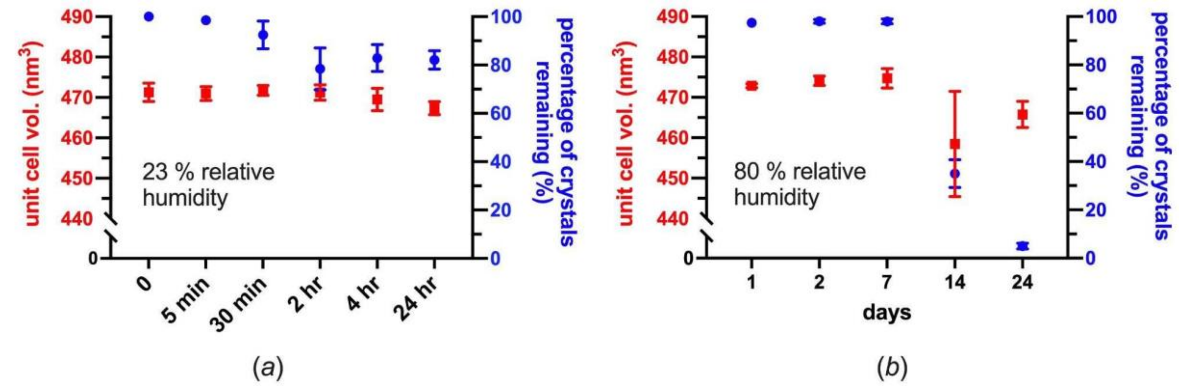


**Figure S1.** Insulin crystal lifetime when left at 23% and 80% relative humidity. Hundreds of 30 µm insulin crystals were loaded onto 3 µm COC (Cyclic Olefin Copolymer) supports and sealed with 13 µm COP (Cyclic Olefin Polymer). The crystals were left at 24 °C for given periods of time. (***a***) and (***b***) show how the unit cell and crystal number change when the supports were either stored at ambient room humidity 23% and 80%, respectively.

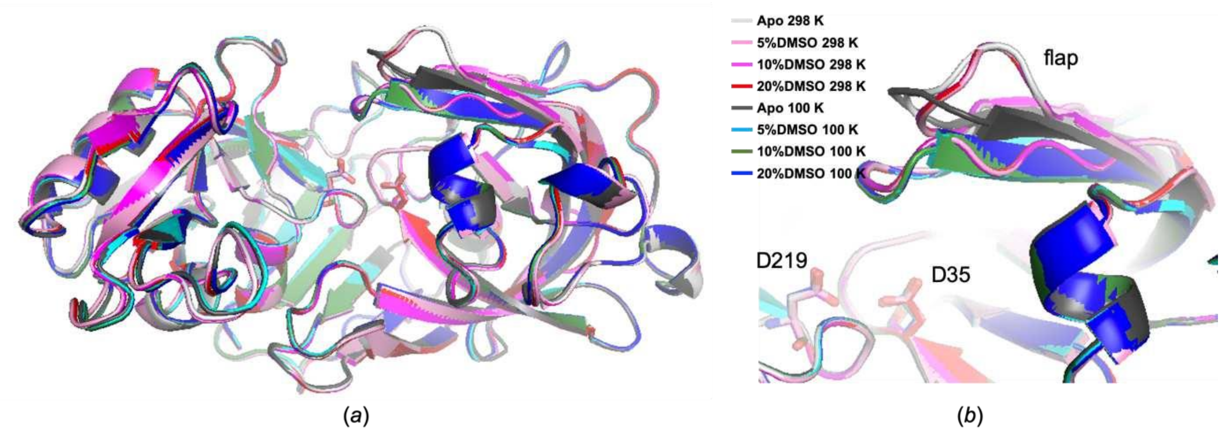


**Figure S2.** Comparison of the EP structures without TL00150 in a series concentration of DMSO at 100 K and 298 K. **(*a*)** Overlay of overall structures. **(*b*)** Enlarged view of the EP active site and flap domain. The EP with 5%, 10%, 20% (*v/v*) DMSO and EP apo, both at 100 K and 298 K are indicated in the panel **(*b*).** From residues 1 to 330 of the EP, the RMSD (Root Mean Square Deviation) for structures of 5%, 10%, and 20% DMSO at 298 K to apo structure at 298 K are 0.065 Å, 0.054 Å, and 0.050 Å, respectively. The RMSD for the same range of residues in structures with 5%, 10%, and 20% DMSO at 100 K to apo structure at 100K are 0.102 Å, 0.121 Å, and 0.126 Å, respectively.

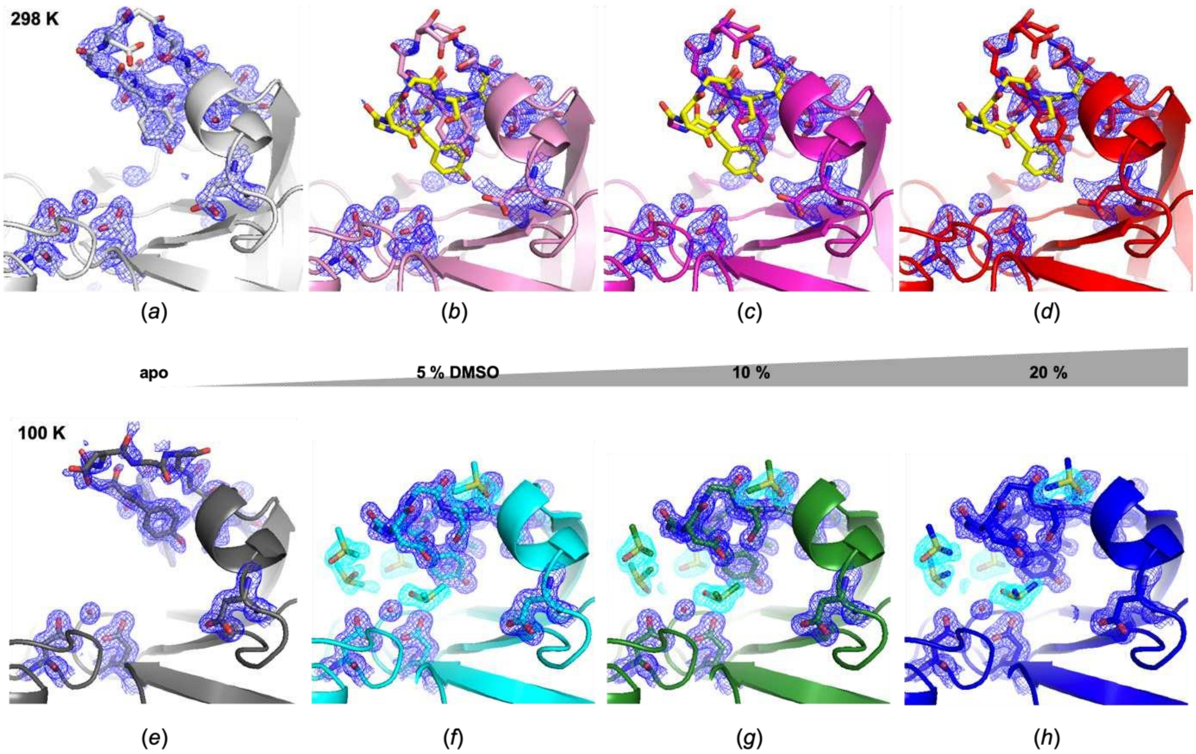


**Figure S3.** 2$F_o$-$F_c$ electron density maps of the EP structures without TL00150 with an increasing concentration of DMSO at 100 K and 298 K. The 2$F_o$-$F_c$ electron density maps contoured at 1σ around the active site and flap domain (residues S76 to S86) of EP structures are shown. Maps of the EP apo, EP structure with 5% (v/v) DMSO, with 10% (v/v) DMSO and with 20% (v/v) DMSO at 298 K are shown in ***(a)***, ***(b)***, ***(c)*** and ***(d)***, respectively. Maps of the EP apo, EP structure with 5%, 10% and 20% (v/v) DMSO at 100 K are shown in ***(e)***, ***(f)***, ***(g)*** and ***(h)***, respectively. The color code for the structures is the same as in Fig. S2. The alternative conformations of RT EP structures are highlighted in yellow. The 2$F_o$-$F_c$ meshes are shown in blue and contoured to 1σ for the flap domain, D35, D119 and D219 and water molecules. The 2$F_o$-$F_c$ meshes for the DMSO molecules are colored in cyan.

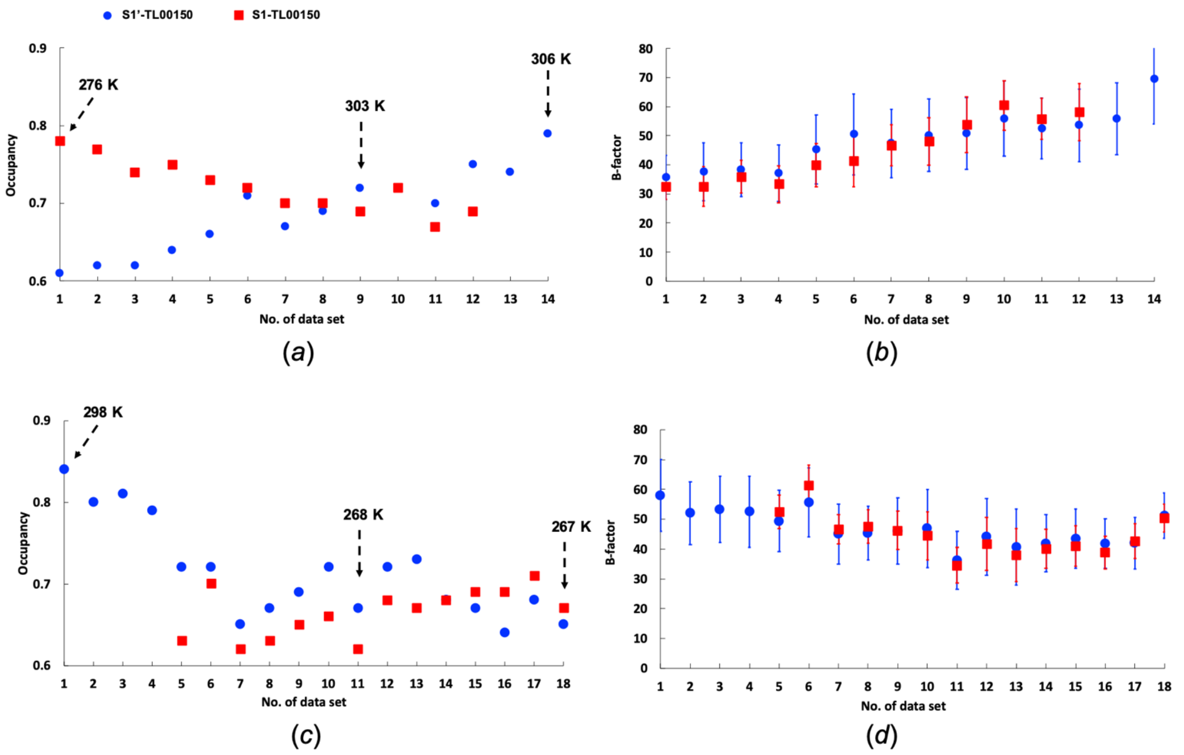


**Figure S4.** Comparison of the occupancy and B-factors of TL00150 in the EP structures. ***(a)*** Occupancy and (***b***) average B-factors of TL00150 for the temperatureramp-up experiment**. (*c*)** Occupancy and (***d***) average B-factors of TL00150 for the temperature ramp-down experiment**.** The temperatures of 276 K, 303 K, and 306 K are indicated in ***(a)*** and 298 K, 268 K and 267 K are indicated in **(*c*)**. The temperatures discussed here were measured from the sample by infrared camera (details describedin §2.3).

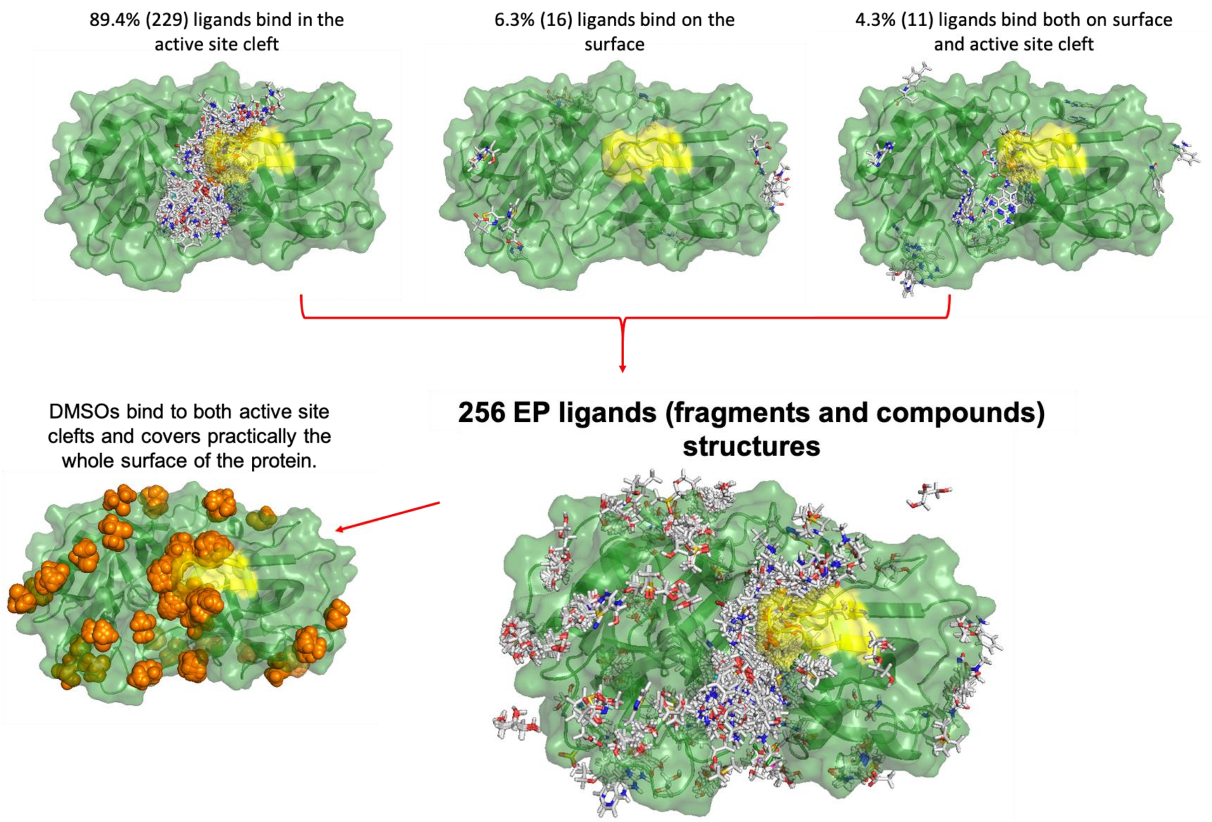


**Figure S5.** Comparison of 256 EP ligand structures. The 256 structures downloaded from the PDB (ref. Table S6) were aligned and classified. The EP structures are shown as surface representation with green color and flap domain is highlighted in yellow. The ligands are shown in stick representation. The DMSOs are highlighted in sphere representation with orange color. Depicted ligand hydrogens were added using the Schrödinger Preparation Wizard (Schrödinger Release 2021-1: Maestro, Schrödinger, LLC, New York, NY, 2021) without energy minimization to enable pose comparisons using Shape Screening with typed atoms.

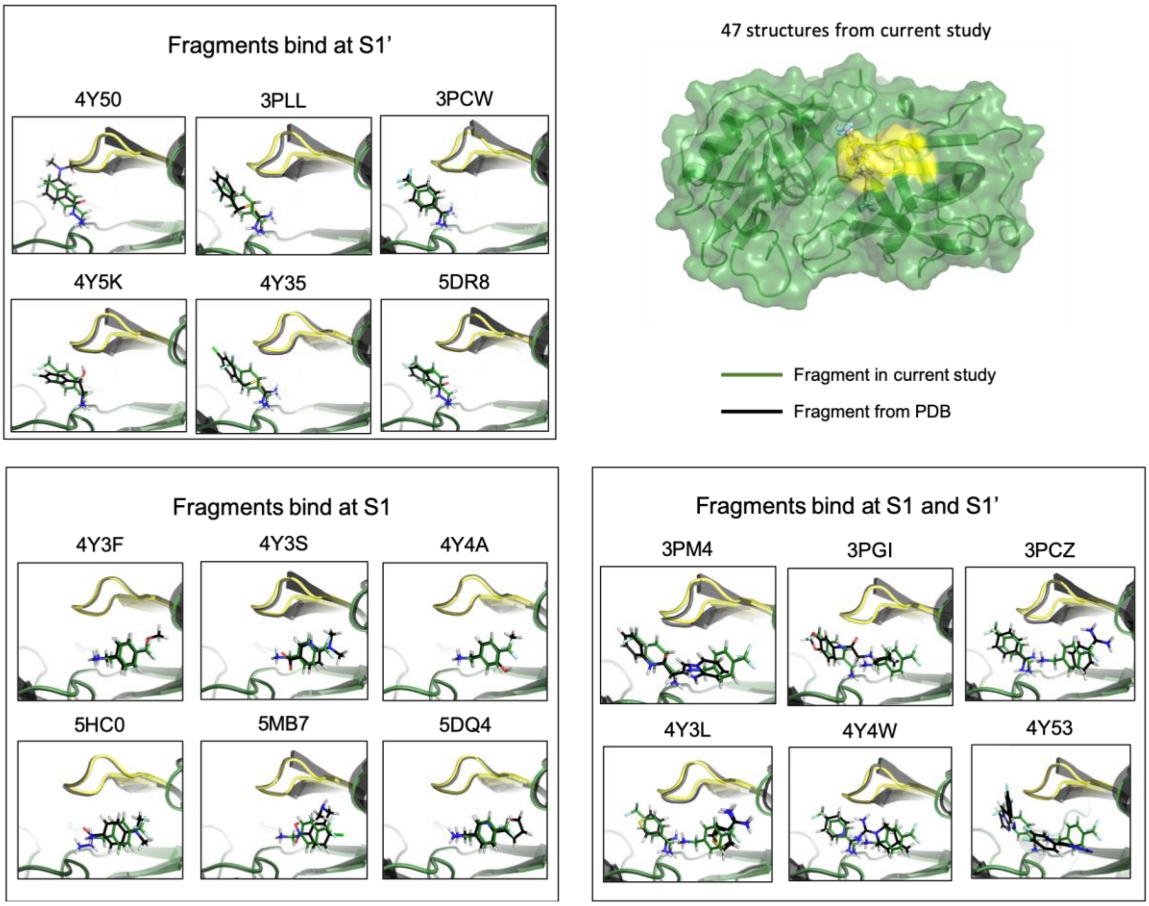


**Figure S6.** Comparison of the fragments from temperature-resolved structures with fragments with similar binding modes taken from the PDB. The enlarged view of the EP active site is shown. The 6 structures were chosen from 256 structures for comparison of S1', S1 and both S1' and S1 binding modes. For TL00150 and other PDB fragments, the fragments are shown in stick representation with green and black color, respectively. In the figures, the PDB codes are shown. Depicted ligand hydrogens were added using the Schrödinger Preparation Wizard (Schrödinger Release 2021-1: Maestro, Schrödinger, LLC, New York, NY, 2021) without energy minimization to enable pose comparisons using Shape Screening with typed atoms.

**Table S1.** Fragment specification

| Fragment name | SMILES description | Chemical structure* |
|---|---|---|
| TL00150 [4(trifluoromethyl)be nzylamine] | NCc1ccc(C(F)(F)F)cc1 | 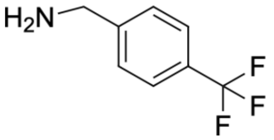  |

*Chemical structure was generated with ChemDraw direct (https://chemdrawdirect.perkinelmer.cloud/js/sample/index.html#)

**Table S2.** Statistic of EP-TL00150 structures at RT of the serial DMSO concentration

| | | 5%DMSO-TL00150 | 8%DMSO-TL00150 | 10%DMSO-TL00150 | 15%DMSO-TL00150 | 20%DMSO-TL00150 | 40%DMSO-TL00150 |
|---|---|---|---|---|---|---|---|
| **PDB code** | | **7QM3** | **7QM4** | **7QM5** | **7QM6** | **7QM7** | **7QM8** |
| Resolution (Å) | | 37.36 - 1.79 (1.85 - 1.79) | 37.39 - 1.79 (1.85 - 1.79) | 35.27 - 1.79 (1.85 - 1.79) | 37.37 - 1.81 (1.88 - 1.81) | 37.37 - 1.79 (1.85 - 1.79) | 37.33 - 1.79 (1.85 - 1.79) |
| Space group | | *P*121 | *P*121 | *P*121 | *P*121 | *P*121 | *P*121 |
| Unit-cell parameters | *a,b,c* (Å) | 45.87 73.91 53.01 | 45.95 74.07 53.32 | 45.91 74.04 53.36 | 45.98 74.09 53.45 | 46.00 74.04 53.55 | 45.96 74.32 53.75 |
| | β (°) | 109.29 | 109.49 | 109.64 | 109.72 | 109.79 | 110.09 |
| Unique reflections | | 31104 (3061) | 31299 (3022) | 31279 (3066) | 30425 (2980) | 31619 (3072) | 31552 (3116) |
| Multiplicity | | 6.93 (6.57) | 6.94 (6.56) | 6.99 (6.67) | 7.02 (6.72) | 6.93 (6.57) | 1.05 (6.67) |
| Completeness (%) | | 98.57 (97.61) | 98.37 (95.00) | 98.46 (96.69) | 98.71 (97.67) | 98.96 (96.21) | 98.43 (97.16) |
| Mean $I/\sigma(I)$ | | 10.30 (1.09) | 13.16 (1.44) | 19.86 (4.08) | 13.07 (1.26) | 9.40 (0.68) | 23.18 (4.28) |
| Wilson B-factor | | 29.47 | 28.96 | 24.36 | 30.84 | 31.41 | 22.10 |
| $R_{meas}$ | | 0.12 (1.39) | 0.19 (1.12) | 0.06 (0.41) | 0.10 (1.38) | 0.14 (2.28) | 0.05 (0.41) |
| CC1/2 | | 0.99 (0.65) | 0.99 (0.73) | 0.99 (0.94) | 0.99 (0.67) | 0.99 (0.37) | 0.99 (0.95) |
| ISa | | 16.88 | 24.73 | 28.95 | 25.96 | 19.10 | 40.09 |
| Reflections used in refinement | | 31089 (3061) | 31296 (3022) | 31276 (3066) | 30420 (2978) | 31576 (3069) | 31548 (3114) |
| Reflections used for $R_{free}$ | | 1555 (153) | 1563 (152) | 1564 (154) | 1520 (149) | 1579 (153) | 1577 (156) |
| $R_{work}/R_{free}$ | | 0.19/0.22 | 0.16/0.19 | 0.15/0.17 | 0.15/0.19 | 0.20/0.23 | 0.14/0.17 |
| No. of atoms | | 2530 | 2530 | 2569 | 2578 | 2614 | 2608 |
| macromolecules | | 2384 | 2384 | 2379 | 2379 | 2379 | 2379 |
| ligands | | 32 | 32 | 32 | 52 | 44 | 36 |
| solvent | | 114 | 114 | 158 | 147 | 191 | 193 |
| Protein residues | | 330 | 330 | 330 | 330 | 330 | 330 |
| RMS(bonds) (Å) | | 0.008 | 0.003 | 0.004 | 0.005 | 0.003 | 0.006 |
| RMS(angles) (°) | | 0.96 | 0.61 | 0.72 | 0.78 | 0.59 | 0.83 |
| Ramachandran favored (%) | | 99.09 | 99.09 | 99.09 | 99.09 | 99.09 | 98.78 |
| Ramachandran allowed (%) | | 0.91 | 0.91 | 0.91 | 0.91 | 0.91 | 1.22 |
| Ramachandran outliers (%) | | 0.00 | 0.00 | 0.00 | 0.00 | 0.00 | 0.00 |
| Clashscore | | 1.28 | 1.70 | 1.28 | 1.27 | 0.64 | 0.85 |
| Average B-factor (Å$^2$) | | 34.57 | 34.19 | 29.67 | 33.14 | 35.05 | 25.99 |
| B-factor macromolecules (Å$^2$) | | 34.28 | 33.55 | 28.60 | 32.08 | 33.97 | 24.81 |
| B-factor ligands (Å$^2$) | | 48.24 | 59.52 | 60.43 | 57.82 | 50.25 | 46.25 |
| B-factor solvent (Å$^2$) | | 36.86 | 40.45 | 39.49 | 41.68 | 44.99 | 36.76 |

*Statistics for the highest-resolution shell are shown in parentheses.
**Data processing statistics are reported with Friedel pairs merged.

**Table S3.** Statistic of EP apo structures, EP structures without TL00150 with an increasing concentration of DMSO and EP structure with TL00150 at both 298 K and100 K

| | apo-298K | 5%DMSO-298K | 10%DMSO-298K | 20%DMSO-298K | apo-100K | 5%DMSO-100K | 10%DMSO-100K | 20%DMSO-100K | 10% DMSO -TL00150-100K |
|---|---|---|---|---|---|---|---|---|---|
| **PDB code** | **7QLY** | **7QLZ** | **7QM0** | **7QM1** | **7QLU** | **7QLV** | **7QLW** | **7QLX** | **7QLT** |
| Resolution (Å) | 37.37 - 1.79 (1.85 - 1.79) | 35.29 - 1.79 (1.85 - 1.79) | 43.26 - 1.79 (1.86 - 1.79) | 35.41 - 1.79 (1.85 - 1.79) | 35.13 - 1.41 (1.46 - 1.41) | 35.15 - 1.41 (1.46 - 1.41) | 35.13 - 1.41 (1.46 - 1.41) | 35.12 - 1.39 (1.44 - 1.39) | 34.96 - 1.39 (1.44 - 1.39) |
| Space group | $P_12_1$ | $P_12_1$ | $P_12_1$ | $P_12_1$ | $P_12_1$ | $P_12_1$ | $P_12_1$ | $P_12_1$ | $P_12_1$ |
| Unit-cell parameters *a,b,c* (Å) | 45.97 74.17 53.54 | 45.97 74.12 53.37 | 45.98 74.23 53.64 | 45.96 74.26 53.65 | 45.68 72.46 53.38 | 45.53 73.51 53.29 | 45.47 73.69 53.28 | 45.45 73.72 53.29 | 45.33 73.47 52.99 |
| Unit-cell parameters β (°) | 109.78 | 109.56 | 109.81 | 109.90 | 110.35 | 110.20 | 110.08 | 110.07 | 109.9 |
| Unique reflections | 31529 (3095) | 31333 (2855) | 31331 (2906) | 31452 (3087) | 60448 (5856) | 63364 (6284) | 62760 (6161) | 65999 (6555) | 65469 (6412) |
| Multiplicity | 6.84 (6.52) | 6.94 (6.48) | 7.04 (6.70) | 7.00 (6.63) | 6.86 (6.91) | 6.77 (6.63) | 6.89 (6.91) | 6.61 (6.52) | 6.8 (6.53) |
| Completeness (%) | 98.70 (97.30) | 98.37 (89.92) | 98.14 (91.04) | 98.32 (97.14) | 96.20 (93.26) | 99.87 (99.37) | 98.71 (97.13) | 99.29 (99.08) | 99.76 (98.13) |
| Mean $I/\sigma(I)$ | 7.58 (0.97) | 6.55 (0.32) | 12.02 (0.73) | 10.33 (0.32) | 11.10 (1.06) | 15.48 (1.34) | 20.03 (1.79) | 19.86 (3.78) | 21.99 (2.35) |
| Wilson B-factor | 30.41 | 33.53 | 37.36 | 34.17 | 17.15 | 17.28 | 17.08 | 13.09 | 15.41 |
| $R_{meas}$ | 0.16 (2.10) | 0.18 (4.04) | 0.10 (1.87) | 0.13 (3.53) | 0.14 (1.88) | 0.08 (1.55) | 0.06 (1.19) | 0.06 (0.52) | 0.06 (0.87) |
| $CC_{1/2}$ | 0.99 (0.42) | 0.99 (0.14) | 0.99 (0.40) | 0.99 (0.17) | 0.99 (0.60) | 0.99 (0.73) | 0.99 (0.81) | 0.99 (0.94) | 0.99 (0.88) |
| ISa | 8.01 | 11.41 | 32.58 | 26.13 | 14.23 | 21.31 | 28.43 | 21.65 | 29.23 |
| Reflections used in refinement | 31514 (3094) | 31324 (2855) | 31325 (2906) | 31451 (3087) | 60426 (5856) | 63331 (6270) | 62738 (6151) | 65862 (6548) | 65442 (6399) |
| Reflections used for $R_{free}$ | 1576 (155) | 1564 (140) | 1566 (146) | 1572 (154) | 3021 (293) | 3166 (314) | 3137 (309) | 3296 (327) | 3272 (320) |
| $R_{work}/R_{free}$ | 0.19/0.22 | 0.18/0.20 | 0.16/0.19 | 0.16/0.19 | 0.20/0.23 | 0.19/0.22 | 0.17/0.19 | 0.20/0.23 | 0.18/0.20 |
| No. of atoms | 2564 | 2573 | 2585 | 2585 | 2998 | 2982 | 2980 | 2977 | 2938 |
| macromolecules | 2376 | 2448 | 2448 | 2448 | 2379 | 2382 | 2382 | 2382 | 2385 |
| ligands | 0 | 0 | 0 | 0 | 12 | 36 | 36 | 36 | 48 |
| solvent | 188 | 125 | 137 | 137 | 607 | 564 | 562 | 559 | 505 |
| Protein residues | 330 | 330 | 330 | 330 | 330 | 330 | 330 | 330 | 330 |
| RMS(bonds) (Å) | 0.002 | 0.003 | 0.003 | 0.002 | 0.007 | 0.012 | 0.010 | 0.007 | 0.009 |
| RMS(angles) (°) | 0.61 | 0.64 | 0.64 | 0.62 | 0.96 | 1.27 | 1.13 | 0.98 | 1.09 |
| Ramachandran favored (%) | 99.39 | 98.48 | 98.78 | 98.78 | 97.87 | 98.78 | 98.48 | 98.48 | 98.78 |
| Ramachandran allowed (%) | 0.61 | 1.52 | 1.22 | 1.22 | 2.13 | 1.22 | 1.52 | 1.52 | 1.22 |
| Ramachandran outliers (%) | 0.00 | 0.00 | 0.00 | 0.00 | 0.00 | 0.00 | 0.00 | 0.00 | 0.00 |
| Clashscore | 1.08 | 1.69 | 1.05 | 1.26 | 1.07 | 1.27 | 2.33 | 1.91 | 1.05 |
| Average B-factor ($Å^2$) | 34.51 | 39.24 | 38.34 | 38.34 | 20.65 | 19.89 | 19.77 | 16.72 | 19.51 |
| B-factor macromolecules ($Å^2$) | 33.89 | 39.03 | 38.06 | 38.00 | 18.03 | 16.91 | 16.51 | 13.81 | 16.63 |
| B-factor ligands ($Å^2$) | - | - | - | - | 32.83 | 35.70 | 35.40 | 29.38 | 30.52 |
| B-factor solvent ($Å^2$) | 42.42 | 43.30 | 43.32 | 44.35 | 30.66 | 31.45 | 32.59 | 28.31 | 32.06 |

*Statistics for the highest-resolution shell are shown in parentheses.

**Data processing statistics are reported with Friedel pairs merged.

**Table S4.** Statistic of EP-TL00150 structures of the temperature ramp-up

| | | dataset1 | dataset2 | dataset3 | dataset4 | dataset5 | dataset6 | dataset7 | dataset8 | dataset9 | dataset10 | dataset11 | dataset12 | dataset13 | dataset14 |
|---|---|---|---|---|---|---|---|---|---|---|---|---|---|---|---|
| **PDB colde** | | **7QMR** | **7QMS** | **7QMT** | **7QMU** | **7QMV** | **7QMW** | **7QMX** | **7QMY** | **7QMZ** | **7QN0** | **7QN1** | **7QN2** | **7QN3** | **7QN4** |
| Temperature (K) | | 276 | 280 | 282 | 286 | 289 | 292 | 295 | 298 | 301 | 303 | 305 | 305 | 306 | 306 |
| Resolution (Å) | | 37.26 - 1.79 | 35.37 - 1.79 | 37.29 - 1.79 | 37.29 - 1.79 | 35.37 - 1.79 | 35.37 - 1.79 | 40.24 - 1.79 | 37.37 - 1.79 | 37.39 - 1.79 | 37.40 - 1.79 | 37.41 - 1.79 | 37.41 - 1.79 | 35.29 - 1.79 | 35.25 - 1.79 |
| | | (1.85 - 1.79) | (1.85 - 1.79) | (1.85 - 1.79) | (1.85 - 1.79) | (1.85 - 1.79) | (1.85 - 1.79) | (1.85 - 1.79) | (1.85 - 1.79) | (1.85 - 1.79) | (1.85 - 1.79) | (1.85 - 1.79) | (1.86 -1.79) | (1.85 - 1.79) | (1.85 - 1.79) |
| Space group | | $P_12_1$ | $P_12_1$ | $P_12_1$ | $P_12_1$ | $P_12_1$ | $P_12_1$ | $P_12_1$ | $P_12_1$ | $P_12_1$ | $P_12_1$ | $P_12_1$ | $P_12_1$ | $P_12_1$ | $P_12_1$ |
| Unit-cell parameters | *a*,*b*,*c* (Å) | 45.85 74.17 | 45.87 74.15 | 45.88 74.14 | 45.89 74.13 | 45.92 74.13 | 45.94 74.13 | 45.97 74.13 | 45.98 74.13 | 46.00 74.14 | 46.00 74.13 | 46.01 74.13 | 46.00 74.11 | 46.01 74.10 | 46.02 74.12 |
| | | 53.65 | 53.64 | 53.62 | 53.62 | 53.59 | 53.58 | 53.55 | 53.53 | 53.49 | 53.44 | 53.42 | 53.36 | 53.31 | 53.21 |
| | β (°) | 109.99 | 109.95 | 109.92 | 109.94 | 109.88 | 109.8 | 109.80 | 109.78 | 109.72 | 109.67 | 109.65 | 109.60 | 109.54 | 109.42 |
| Unique reflections | | 31420 (3101) | 31415 (3106) | 31420 (3096) | 31436 (3097) | 31445 (3101) | 31442 (3096) | 31465 (3096) | 31444 (3068) | 31445 (3066) | 31443 (3079) | 31438 (3080) | 31391 (3083) | 31376 (3060) | 30983 (2690) |
| Multiplicity | | 7.04 (6.62) | 7.01 (6.61) | 7.01 (6.64) | 7.02 (6.61) | 7.00 (6.61) | 7.00 (6.61) | 6.99 (6.59) | 6.98 (6.60) | 6.99 (6.59) | 6.99 (6.57) | 6.98 (6.57) | 6.99 (6.59) | 6.99 (6.57) | 7.00 (6.61) |
| Completeness (%) | | 98.58 (96.94) | 98.59 (97.03) | 98.56 (96.84) | 98.58 (96.81) | 98.60 (97.15) | 98.55 (96.78) | 98.56 (96.99) | 98.50 (96.24) | 98.51 (96.17) | 98.54 (96.70) | 98.58 (96.89) | 98.61 (97.16) | 98.54 (96.50) | 97.36 (84.94) |
| Mean $I/\sigma(I)$ | | 11.9 (1.62) | 14.00 (2.33) | 8.93 (0.88) | 13.59 (2.39) | 12.30 (1.54) | 12.64 (1.54) | 12.23 (1.10) | 12.64 (1.33) | 12.59 (1.22) | 12.27 (0.95) | 11.95 (0.92) | 12.50 (1.13) | 12.09 (0.95) | 9.80 (0.28) |
| Wilson B-factor | | 23.42 | 22.84 | 24.25 | 22.53 | 25.56 | 26.16 | 29.61 | 28.98 | 30.58 | 32.96 | 33.02 | 31.96 | 32.94 | 40.55 |
| $R_{meas}$ | | 0.12 (0.98) | 0.10 (0.70) | 0.15 (1.67) | 0.10 (0.70) | 0.11 (1.03) | 0.10 (1.07) | 0.10 (1.54) | 0.10 (1.27) | 0.09 (1.37) | 0.10 (1.73) | 0.10 (1.76) | 0.09 (1.37) | 0.10 (1.66) | 0.12 (3.88) |
| $CC_{1/2}$ | | 0.99 (0.76) | 0.99 (0.85) | 0.99 (0.65) | 0.99 (0.85) | 0.99 (0.73) | 0.99 (0.70) | 0.99 (0.59) | 0.99 (0.61) | 0.99 (0.56) | 0.99 (0.46) | 0.99 (0.44) | 0.99 (0.54) | 0.99 (0.47) | 0.99 (0.12) |
| ISa | | 19.47 | 20.01 | 18.72 | 18.37 | 19.63 | 19.60 | 20.37 | 18.62 | 20.11 | 21.27 | 20.25 | 22.20 | 23.20 | 23.28 |
| Reflections used in refinement | | 31416 (3100) | 31413 (3106) | 31416 (3096) | 31432 (3097) | 31441 (3100) | 31438 (3094) | 31461 (3095) | 31439 (3068) | 31440 (3066) | 31436 (3079) | 31431 (3080) | 31386 (3083) | 31370 (3060) | 30972 (2690) |
| Reflections used for $R_{free}$ | | 1633 (148) | 1639 (156) | 1644 (162) | 1634 (150) | 1634 (151) | 1633 (149) | 1636 (148) | 1635 (148) | 1634 (147) | 1636 (148) | 1634 (147) | 1632 (146) | 1631 (144) | 1614 (130) |
| $R_{work}/R_{free}$ | | 0.17/0.20 | 0.17/0.19 | 0.16/0.19 | 0.16/0.19 | 0.17/0.19 | 0.16/0.18 | 0.17/0.20 | 0.16/0.19 | 0.16/0.18 | 0.17/0.19 | 0.17/0.19 | 0.16/0.19 | 0.16/0.19 | 0.16/0.20 |
| No. of atoms | | 2661 | 2650 | 2638 | 2656 | 2634 | 2604 | 2589 | 2578 | 2582 | 2561 | 2552 | 2547 | 2531 | 2501 |
| macromolecules | | 2379 | 2379 | 2379 | 2379 | 2379 | 2379 | 2379 | 2379 | 2379 | 2382 | 2379 | 2379 | 2379 | 2379 |
| ligands | | 52 | 52 | 52 | 52 | 52 | 52 | 52 | 52 | 52 | 52 | 52 | 52 | 40 | 40 |
| solvent | | 230 | 219 | 207 | 225 | 203 | 173 | 158 | 147 | 151 | 127 | 121 | 116 | 112 | 82 |
| Protein residues | | 330 | 330 | 330 | 330 | 330 | 330 | 330 | 330 | 330 | 330 | 330 | 330 | 330 | 330 |
| RMS(bonds) (Å) | | 0.006 | 0.003 | 0.006 | 0.006 | 0.003 | 0.003 | 0.005 | 0.014 | 0.005 | 0.005 | 0.005 | 0.016 | 0.015 | 0.010 |
| RMS(angles) (°) | | 0.84 | 0.62 | 0.82 | 0.82 | 0.64 | 0.68 | 0.76 | 1.26 | 0.79 | 0.77 | 0.79 | 1.45 | 1.34 | 1.00 |
| Ramachandran favored (%) | | 98.78 | 99.09 | 98.78 | 99.09 | 98.78 | 99.09 | 99.09 | 98.17 | 98.78 | 98.78 | 98.48 | 99.09 | 98.78 | 97.87 |
| Ramachandran allowed (%) | | 1.22 | 0.91 | 1.22 | 0.91 | 1.22 | 0.91 | 0.91 | 1.83 | 1.22 | 1.22 | 1.52 | 0.91 | 1.22 | 2.13 |
| Ramachandran outliers (%) | | 0.00 | 0.00 | 0.00 | 0.00 | 0.00 | 0.00 | 0.00 | 0.00 | 0.00 | 0.00 | 0.00 | 0.00 | 0.00 | 0.00 |
| Clashscore | | 1.27 | 1.27 | 1.48 | 1.27 | 1.70 | 1.70 | 1.27 | 1.48 | 1.48 | 1.69 | 1.48 | 1.48 | 1.70 | 1.49 |
| Average B-factor ($Å^2$) | | 26.59 | 25.96 | 27.30 | 25.70 | 29.31 | 29.85 | 33.51 | 32.55 | 33.73 | 37.38 | 37.23 | 35.47 | 38.38 | 48.45 |
| B-factor macromolecules ($Å^2$) | | 25.51 | 24.86 | 26.19 | 24.48 | 28.18 | 28.74 | 32.63 | 31.70 | 32.74 | 36.59 | 36.50 | 34.72 | 37.80 | 48.04 |
| B-factor ligands ($Å^2$) | | 36.55 | 38.15 | 40.02 | 38.27 | 44.28 | 48.72 | 48.28 | 50.33 | 53.40 | 58.01 | 54.60 | 56.16 | 57.49 | 71.89 |
| B-factor solvent ($Å^2$) | | 35.45 | 35.00 | 36.90 | 35.72 | 38.73 | 39.31 | 41.96 | 40.03 | 42.43 | 43.90 | 43.98 | 41.55 | 43.90 | 48.77 |

*Statistics for the highest-resolution shell are shown in parentheses.

**Data processing statistics are reported with Friedel pairs merged.

**Table S5.** Statistic of EP-TL00150 structures of the temperature ramp-down

| | dataset1 | dataset2 | dataset3 | dataset4 | dataset5 | dataset6 | dataset7 | dataset8 | dataset9 | dataset10 | dataset11 | dataset12 | dataset13 | dataset14 | dataset15 | dataset16 | dataset17 | dataset18 |
|---|---|---|---|---|---|---|---|---|---|---|---|---|---|---|---|---|---|---|
| **PDB code** | **7QM9** | **7QMA** | **7QMB** | **7QMC** | **7QMD** | **7QME** | **7QMF** | **7QMG** | **7QMH** | **7QMI** | **7QMJ** | **7QMK** | **7QML** | **7QMM** | **7QMN** | **7QMO** | **7QMP** | **7QMQ** |
| Temperature (K) | 298 | 296 | 293 | 290 | 287 | 284 | 281 | 278 | 276 | 273 | 271 | 270 | 268 | 267 | 266 | 266 | 267 | 267 |
| Resolution (Å) | 39.86 - 1.79 (1.85 - 1.79) | 35.11 - 1.79 (1.85 - 1.79) | 35.14 - 1.79 (1.85 - 1.79) | 37.36 - 1.79 (1.85 - 1.79) | 37.37 - 1.79 (1.86 - 1.79) | 35.23 - 1.79 (1.86 - 1.79) | 37.36 - 1.79 (1.86 - 1.79) | 35.25 - 1.79 (1.86 - 1.79) | 37.31 - 1.79 (1.85 - 1.79) | 35.26 - 1.7 (1.86 - 1.79) | 37.28 - 1.79 (1.86 - 1.79) | 35.24 - 1.79 (1.86 - 1.79) | 37.27 - 1.79 (1.85 - 1.79) | 43.14 - 1.79 (1.85 - 1.79) | 36.96 - 1.79 (1.85 - 1.79) | 37.02 - 1.79 (1.85 - 1.79) | 37.00 - 1.79 (1.85 - 1.79) | 40.04 - 1.79 (1.86 - 1.79) |
| Space group | *P*121 | *P*121 | *P*121 | *P*121 | *P*121 | *P*121 | *P*121 | *P*121 | *P*121 | *P*121 | *P*121 | *P*121 | *P*121 | *P*121 | *P*121 | *P*121 | *P*121 | *P*121 |
| Unit-cell parameters *a,b,c* (Å) | 45.86 73.90 52.96 | 45.87 73.93 53.04 | 45.89 73.96 53.11 | 45.89 73.96 53.18 | 45.90 74.01 53.31 | 45.92 74.05 53.34 | 45.92 74.07 53.48 | 45.88 74.02 53.40 | 45.85 73.99 53.42 | 45.86 74.01 53.46 | 45.82 73.99 53.46 | 45.81 73.97 53.45 | 45.81 73.98 53.48 | 45.80 73.99 53.51 | 45.75 73.92 53.43 | 45.73 73.91 53.39 | 45.79 74.00 53.50 | 45.77 73.95 53.40 |
| β (°) | 109.19 | 109.22 | 109.25 | 109.32 | 109.42 | 109.37 | 109.54 | 109.52 | 109.57 | 109.59 | 109.64 | 109.52 | 109.63 | 109.65 | 109.70 | 109.72 | 109.7 | 109.6 |
| Unique reflections | 31109 (3070) | 31147 (3065) | 31187 (3062) | 31201 (3056) | 31270 (3070) | 31222 (3084) | 31662 (3116) | 31685 (3134) | 31686 (3140) | 31678 (3136) | 31676 (3146) | 31657 (3143) | 31652 (3134) | 31671 (3121) | 31611 (3163) | 31549 (3144) | 31678 (3150) | 31191 (2782) |
| Multiplicity | 6.96 (6.56) | 6.94 (6.56) | 6.96 (6.55) | 6.93 (6.57) | 6.91 (6.56) | 6.94 (6.55) | 6.38 (6.70) | 6.83 (6.38) | 6.83 (6.49) | 6.83 (6.41) | 6.81 (6.40) | 6.81 (6.39) | 6.81 (6.37) | 6.72 (6.33) | 6.80 (6.36) | 6.79 (6.38) | 6.80 (6.40) | 6.82 (6.42) |
| Completeness (%) | 98.61 (97.62) | 98.59 (97.46) | 98.60 (97.36) | 98.57 (96.83) | 98.58 (97.06) | 98.68 (97.75) | 99.65 (98.39) | 99.80 (98.77) | 99.84 (99.49) | 99.85 (99.33) | 99.86 (99.53) | 99.85 (99.52) | 99.78 (98.80) | 99.74 (98.39) | 99.92 (99.91) | 99.86 (99.87) | 99.73 (99.06) | 98.63 (88.32) |
| Mean $I/\sigma(I)$ | 10.38 (0.65) | 13.55 (1.29) | 12.21 (1.06) | 12.73 (1.15) | 11.45 (0.93) | 8,35 (0.47) | 9.43 (1.04) | 11.39 (1.16) | 12.52 (1.67) | 13.57 (2.37) | 16.12 (3.42) | 15.80 (3.26) | 15.07 (3.18) | 11.87 (2.07) | 11.70 (1.59) | 7.89 (0.92) | 6.70 (0.57) | 5.71 (0.17) |
| Wilson B-factor | 33.79 | 30.26 | 30.92 | 31.01 | 31.19 | 33.29 | 28.64 | 30.01 | 27.10 | 25.53 | 22.52 | 22.46 | 22.02 | 23.98 | 25.39 | 26.35 | 28.66 | 35.08 |
| $R_{meas}$ | 0.12 (2.21) | 0.09 (1.35) | 0.10 (1.60) | 0.09 (1.51) | 0.11 (1.76) | 0.15 (3.27) | 0.15 (1.75) | 0.10 (1.49) | 0.10 (1.04) | 0.09 (0.70) | 0.08 (0.48) | 0.08 (0.50) | 0.09 (0.53) | 0.13 (0.83) | 0.13 (0.95) | 0.23 (1.69) | 0.28 (2.57) | 0.26 (4.78) |
| $CC_{1/2}$ | 0.99 (0.36) | 0.99 (0.61) | 0.99 (0.52) | 0.99 (0.54) | 0.99 (0.44) | 0.99 (0.24) | 0.99 (0.51) | 0.99 (0.54) | 0.99 (0.69) | 0.99 (0.83) | 0.99 (0.91) | 0.99 (0.91) | 0.99 (0.90) | 0.99 (0.83) | 0.99 (0.80) | 0.99 (0.63) | 0.99 (0.41) | 0.99 (0.14) |
| ISa | 21.03 | 24.99 | 22.31 | 22.23 | 21.23 | 16.29 | 17.12 | 17.65 | 17.92 | 16.77 | 19.94 | 20.02 | 18.42 | 18.71 | 20.66 | 16.70 | 17.72 | 17.65 |
| Reflections used in refinement | 31094 (3070) | 31136 (3065) | 31167 (3062) | 31195 (3056) | 31251 (3070) | 31208 (3084) | 31646 (3116) | 31678 (3133) | 31672 (3140) | 31665 (3136) | 31660 (3144) | 31639 (3140) | 31641 (3132) | 31664 (3120) | 31605 (3162) | 31532 (3142) | 31648 (3148) | 31180 (2782) |
| Reflections used for $R_{free}$ | 1540 (160) | 1541 (160) | 1542 (160) | 1545 (159) | 1547 (160) | 1545 (158) | 1567 (164) | 1571 (165) | 1569 (165) | 1569 (165) | 1567 (164) | 1566 (165) | 1568 (166) | 1568 (166) | 1568 (164) | 1565 (164) | 1569 (168) | 1552 (149) |
| $R_{work}/R_{free}$ | 0.20/0.23 | 0.19/0.21 | 0.20/0.22 | 0.19/0.22 | 0.19/0.21 | 0.19/0.23 | 0.19/0.22 | 0.19/0.22 | 0.18/0.22 | 0.18/0.21 | 0.18/0.20 | 0.16/0.19 | 0.17/0.19 | 0.17/0.20 | 0.17/0.20 | 0.19/0.22 | 0.21/0.25 | 0.21/0.24 |
| No. of atoms | 2533 | 2546 | 2543 | 2549 | 2565 | 2564 | 2596 | 2605 | 2622 | 2610 | 2664 | 2657 | 2673 | 2659 | 2652 | 2631 | 2615 | 2585 |
| macromolecules | 2384 | 2384 | 2384 | 2384 | 2379 | 2379 | 2379 | 2379 | 2379 | 2379 | 2379 | 2379 | 2379 | 2379 | 2379 | 2379 | 2379 | 2379 |
| ligands | 36 | 36 | 36 | 36 | 48 | 48 | 48 | 48 | 48 | 48 | 48 | 48 | 48 | 48 | 48 | 48 | 48 | 48 |
| solvent | 113 | 126 | 123 | 129 | 138 | 137 | 169 | 178 | 195 | 183 | 237 | 230 | 246 | 232 | 225 | 204 | 188 | 158 |
| Protein residues | 330 | 330 | 330 | 330 | 330 | 330 | 330 | 330 | 330 | 330 | 330 | 330 | 330 | 330 | 330 | 330 | 330 | 330 |
| RMS(bonds) (Å) | 0.006 | 0.003 | 0.003 | 0.005 | 0.002 | 0.007 | 0.005 | 0.006 | 0.005 | 0.004 | 0.004 | 0.006 | 0.004 | 0.004 | 0.004 | 0.002 | 0.005 | 0.003 |
| RMS(angles) (°) | 0.80 | 0.64 | 0.65 | 0.72 | 0.59 | 0.88 | 0.80 | 0.84 | 0.81 | 0.71 | 0.69 | 0.82 | 0.73 | 0.71 | 0.69 | 0.58 | 0.75 | 0.59 |
| Ramachandran favored (%) | 98.78 | 98.48 | 98.78 | 98.78 | 99.09 | 98.78 | 99.09 | 98.78 | 98.48 | 98.48 | 99.09 | 98.48 | 98.78 | 99.09 | 98.78 | 98.78 | 98.78 | 99.09 |
| Ramachandran allowed (%) | 1.22 | 1.52 | 1.22 | 1.22 | 0.91 | 1.22 | 0.91 | 1.22 | 1.52 | 1.52 | 0.91 | 1.52 | 1.22 | 0.91 | 1.22 | 1.22 | 1.22 | 0.91 |
| Ramachandran outliers (%) | 0.00 | 0.00 | 0.00 | 0.00 | 0.00 | 0.00 | 0.00 | 0.00 | 0.00 | 0.00 | 0.00 | 0.00 | 0.00 | 0.00 | 0.00 | 0.00 | 0.00 | 0.00 |
| Clashscore | 1.49 | 1.06 | 1.49 | 1.28 | 1.27 | 1.27 | 0.42 | 0.64 | 1.06 | 1.70 | 0.42 | 0.85 | 0.64 | 0.64 | 1.06 | 1.06 | 0.64 | 0.64 |
| Average B-factor ($Å^2$) | 40.28 | 36.57 | 38.05 | 36.29 | 35.54 | 39.97 | 32.29 | 33.37 | 30.53 | 28.63 | 23.66 | 26.02 | 23.61 | 28.36 | 28.56 | 31.20 | 32.14 | 39.96 |
| B-factor macromolecules ($Å^2$) | 39.84 | 36.01 | 37.40 | 35.73 | 34.91 | 39.47 | 31.41 | 32.52 | 29.40 | 27.61 | 22.32 | 24.51 | 21.99 | 27.12 | 27.38 | 30.36 | 31.43 | 39.32 |
| B-factor ligands ($Å^2$) | 56.14 | 51.48 | 53.60 | 52.55 | 49.69 | 55.56 | 46.28 | 46.20 | 45.56 | 46.45 | 37.76 | 45.53 | 40.60 | 42.64 | 43.55 | 41.70 | 43.06 | 50.79 |
| B-factor solvent ($Å^2$) | 44.50 | 42.98 | 46.12 | 42.06 | 41.45 | 43.28 | 40.72 | 41.39 | 40.58 | 37.24 | 34.22 | 37.57 | 35.99 | 38.23 | 37.77 | 38.54 | 38.37 | 46.29 |

*Statistics for the highest-resolution shell are shown in parentheses.
**Data processing statistics are reported with Friedel pairs merged.

**Table S6.** PDB ID and its corresponding DOI

| PDB ID | PDB DOI |
|---|---|
| 1E5O | 10.2210/pdb1E5O/pdb |
| 1E80 | 10.2210/pdb1E80/pdb |
| 1E81 | 10.2210/pdb1E81/pdb |
| 1E82. | 10.2210/pdb1E82/pdb |
| 1EED | 10.2210/pdb1EED/pdb |
| 1ENT | 10.2210/pdb1ENT/pdb |
| 1EPL | 10.2210/pdb1EPL/pdb |
| 1EPM | 10.2210/pdb1EPM/pdb |
| 1EPN | 10.2210/pdb1EPN/pdb |
| 1EPO | 10.2210/pdb1EPO/pdb |
| 1EPP | 10.2210/pdb1EPP/pdb |
| 1EPQ | 10.2210/pdb1EPQ/pdb |
| 1EPR | 10.2210/pdb1EPR/pdb |
| 1ER8 | 10.2210/pdb1ER8/pdb |
| 1GKT | 10.2210/pdb1GKT/pdb |
| 1GVT | 10.2210/pdb1GVT/pdb |
| 1GVU | 10.2210/pdb1GVU/pdb |
| 1GVV | 10.2210/pdb1GVV/pdb |
| 1GVW | 10.2210/pdb1GVW/pdb |
| 1GVX | 10.2210/pdb1GVX/pdb |
| 1OD1 | 10.2210/pdb1OD1/pdb |
| 1OEW | 10.2210/pdb1OEW/pdb |
| 1OEX | 10.2210/pdb1OEX/pdb |
| 2ER0 | 10.2210/pdb2ER0/pdb |
| 2ER6 | 10.2210/pdb2ER6/pdb |
| 2ER7 | 10.2210/pdb2ER7/pdb |
| 2ER9 | 10.2210/pdb2ER9/pdb |
| 2JJI | 10.2210/pdb2JJI/pdb |
| 2JJJ | 10.2210/pdb2JJJ/pdb |
| 2V00 | 10.2210/pdb2V00/pdb |
| 2VS2 | 10.2210/pdb2VS2/pdb |
| 3ER3 | 10.2210/pdb3ER3/pdb |
| 3ER5 | 10.2210/pdb3ER5/pdb |
| 3LZY | 10.2210/pdb3LZY/pdb |
| 3P1D | 10.2210/pdb3P1D/pdb |
| 3PB5 | 10.2210/pdb3PB5/pdb |
| 3PBD | 10.2210/pdb3PBD/pdb |
| 3PBZ | 10.2210/pdb3PBZ/pdb |
| 3PCW | 10.2210/pdb3PCW/pdb |
| 3PCZ | 10.2210/pdb3PCZ/pdb |
| 3PGI | 10.2210/pdb3PGI/pdb |
| 3PI0 | 10.2210/pdb3PI0/pdb |
| 3PLL | 10.2210/pdb3PLL/pdb |
| 3PM4 | 10.2210/pdb3PM4/pdb |
| 3PMU | 10.2210/pdb3PMU/pdb |
| 3PMY | 10.2210/pdb3PMY/pdb |

| PDB ID | PDB DOI |
|---|---|
| 3PRS | 10.2210/pdb3PRS/pdb |
| 3PSY | 10.2210/pdb3PSY/pdb |
| 3PWW | 10.2210/pdb3PWW/pdb |
| 3Q6Y | 10.2210/pdb3Q6Y/pdb |
| 3T6I | 10.2210/pdb3T6I/pdb |
| 3T7P | 10.2210/pdb3T7P/pdb |
| 3T7Q | 10.2210/pdb3T7Q/pdb |
| 3T7X | 10.2210/pdb3T7X/pdb |
| 3URI | 10.2210/pdb3URI/pdb |
| 3URL | 10.2210/pdb3URL/pdb |
| 3WZ6 | 10.2210/pdb3WZ6/pdb |
| 3WZ7 | 10.2210/pdb3WZ7/pdb |
| 3WZ8 | 10.2210/pdb3WZ8/pdb |
| 4ER1 | 10.2210/pdb4ER1/pdb |
| 4ER2 | 10.2210/pdb4ER2/pdb |
| 4ER4 | 10.2210/pdb4ER4/pdb |
| 4KUP | 10.2210/pdb4KUP/pdb |
| 4L6B | 10.2210/pdb4L6B/pdb |
| 4LAP | 10.2210/pdb4LAP/pdb |
| 4LBT | 10.2210/pdb4LBT/pdb |
| 4LHH | 10.2210/pdb4LHH/pdb |
| 4LP9 | 10.2210/pdb4LP9/pdb |
| 4Y35 | 10.2210/pdb4Y35/pdb |
| 4Y36 | 10.2210/pdb4Y36/pdb |
| 4Y37 | 10.2210/pdb4Y37/pdb |
| 4Y38 | 10.2210/pdb4Y38/pdb |
| 4Y39 | 10.2210/pdb4Y39/pdb |
| 4Y3A | 10.2210/pdb4Y3A/pdb |
| 4Y3D | 10.2210/pdb4Y3D/pdb |
| 4Y3E | 10.2210/pdb4Y3E/pdb |
| 4Y3F | 10.2210/pdb4Y3F/pdb |
| 4Y3G | 10.2210/pdb4Y3G/pdb |
| 4Y3H | 10.2210/pdb4Y3H/pdb |
| 4Y3J | 10.2210/pdb4Y3J/pdb |
| 4Y3L | 10.2210/pdb4Y3L/pdb |
| 4Y3M | 10.2210/pdb4Y3M/pdb |
| 4Y3N | 10.2210/pdb4Y3N/pdb |
| 4Y3P | 10.2210/pdb4Y3P/pdb |
| 4Y3Q | 10.2210/pdb4Y3Q/pdb |
| 4Y3R | 10.2210/pdb4Y3R/pdb |
| 4Y3S | 10.2210/pdb4Y3S/pdb |
| 4Y3T | 10.2210/pdb4Y3T/pdb |
| 4Y3W | 10.2210/pdb4Y3W/pdb |
| 4Y3X | 10.2210/pdb4Y3X/pdb |
| 4Y3Y | 10.2210/pdb4Y3Y/pdb |
| 4Y3Z | 10.2210/pdb4Y3Z/pdb |
| 4Y41 | 10.2210/pdb4Y41/pdb |

| PDB ID | PDB DOI |
|---|---|
| 4Y43 | 10.2210/pdb4Y43/pdb |
| 4Y44 | 10.2210/pdb4Y44/pdb |
| 4Y45 | 10.2210/pdb4Y45/pdb |
| 4Y47 | 10.2210/pdb4Y47/pdb |
| 4Y48 | 10.2210/pdb4Y48/pdb |
| 4Y4A | 10.2210/pdb4Y4A/pdb |
| 4Y4B | 10.2210/pdb4Y4B/pdb |
| 4Y4D | 10.2210/pdb4Y4D/pdb |
| 4Y4E | 10.2210/pdb4Y4E/pdb |
| 4Y4G | 10.2210/pdb4Y4G/pdb |
| 4Y4J | 10.2210/pdb4Y4J/pdb |
| 4Y4T | 10.2210/pdb4Y4T/pdb |
| 4Y4U | 10.2210/pdb4Y4U/pdb |
| 4Y4W | 10.2210/pdb4Y4W/pdb |
| 4Y4X | 10.2210/pdb4Y4X/pdb |
| 4Y4Z | 10.2210/pdb4Y4Z/pdb |
| 4Y50 | 10.2210/pdb4Y50/pdb |
| 4Y51 | 10.2210/pdb4Y51/pdb |
| 4Y53 | 10.2210/pdb4Y53/pdb |
| 4Y54 | 10.2210/pdb4Y54/pdb |
| 4Y56 | 10.2210/pdb4Y56/pdb |
| 4Y57 | 10.2210/pdb4Y57/pdb |
| 4Y58 | 10.2210/pdb4Y58/pdb |
| 4Y5A | 10.2210/pdb4Y5A/pdb |
| 4Y5B | 10.2210/pdb4Y5B/pdb |
| 4Y5C | 10.2210/pdb4Y5C/pdb |
| 4Y5E | 10.2210/pdb4Y5E/pdb |
| 4Y5G | 10.2210/pdb4Y5G/pdb |
| 4Y5K | 10.2210/pdb4Y5K/pdb |
| 4Y5L | 10.2210/pdb4Y5L/pdb |
| 4Y5M | 10.2210/pdb4Y5M/pdb |
| 4Y5N | 10.2210/pdb4Y5N/pdb |
| 4Y5P | 10.2210/pdb4Y5P/pdb |
| 4YCK | 10.2210/pdb4YCK/pdb |
| 4YCT | 10.2210/pdb4YCT/pdb |
| 4YCY | 10.2210/pdb4YCY/pdb |
| 4YD3 | 10.2210/pdb4YD3/pdb |
| 4YD4 | 10.2210/pdb4YD4/pdb |
| 4YD5 | 10.2210/pdb4YD5/pdb |
| 4YD6 | 10.2210/pdb4YD6/pdb |
| 4YD7 | 10.2210/pdb4YD7/pdb |
| 4ZE6 | 10.2210/pdb4ZE6/pdb |
| 4ZEA | 10.2210/pdb4ZEA/pdb |
| 5DPZ | 10.2210/pdb5DPZ/pdb |
| 5DQ1 | 10.2210/pdb5DQ1/pdb |
| 5DQ2 | 10.2210/pdb5DQ2/pdb |
| 5DQ4 | 10.2210/pdb5DQ4/pdb |

| PDB ID | PDB DOI |
|---|---|
| 5DQ5 | 10.2210/pdb5DQ5/pdb |
| 5DR0 | 10.2210/pdb5DR0/pdb |
| 5DR1 | 10.2210/pdb5DR1/pdb |
| 5DR3 | 10.2210/pdb5DR3/pdb |
| 5DR4 | 10.2210/pdb5DR4/pdb |
| 5DR7 | 10.2210/pdb5DR7/pdb |
| 5DR8 | 10.2210/pdb5DR8/pdb |
| 5ER1 | 10.2210/pdb5ER1/pdb |
| 5ER2 | 10.2210/pdb5ER2/pdb |
| 5HCO | 10.2210/pdb5HCO/pdb |
| 5HCT | 10.2210/pdb5HCT/pdb |
| 5IS4 | 10.2210/pdb5IS4/pdb |
| 5ISJ | 10.2210/pdb5ISJ/pdb |
| 5ISK | 10.2210/pdb5ISK/pdb |
| 5J25 | 10.2210/pdb5J25/pdb |
| 5LWR | 10.2210/pdb5LWR/pdb |
| 5LWS | 10.2210/pdb5LWS/pdb |
| 5LWT | 10.2210/pdb5LWT/pdb |
| 5LWU | 10.2210/pdb5LWU/pdb |
| 5MB0 | 10.2210/pdb5MB0/pdb |
| 5MB3 | 10.2210/pdb5MB3/pdb |
| 5MB5 | 10.2210/pdb5MB5/pdb |
| 5MB6 | 10.2210/pdb5MB6/pdb |
| 5MB7 | 10.2210/pdb5MB7/pdb |
| 5OG7 | 10.2210/pdb5OG7/pdb |
| 5OJE | 10.2210/pdb5OJE/pdb |
| 5QB5 | 10.2210/pdb5QB5/pdb |
| 5QB6 | 10.2210/pdb5QB6/pdb |
| 5QB7 | 10.2210/pdb5QB7/pdb |
| 5QB8 | 10.2210/pdb5QB8/pdb |
| 5QB9 | 10.2210/pdb5QB9/pdb |
| 5QBA | 10.2210/pdb5QBA/pdb |
| 5QBB | 10.2210/pdb5QBB/pdb |
| 5QBC | 10.2210/pdb5QBC/pdb |
| 5QBD | 10.2210/pdb5QBD/pdb |
| 5QBE | 10.2210/pdb5QBE/pdb |
| 5QBF | 10.2210/pdb5QBF/pdb |
| 5QBG | 10.2210/pdb5QBG/pdb |
| 5QBH | 10.2210/pdb5QBH/pdb |
| 5QBI | 10.2210/pdb5QBI/pdb |
| 5QBJ | 10.2210/pdb5QBJ/pdb |
| 5QBK | 10.2210/pdb5QBK/pdb |
| 5QBL | 10.2210/pdb5QBL/pdb |
| 5QBM | 10.2210/pdb5QBM/pdb |
| 5QBN | 10.2210/pdb5QBN/pdb |
| 5QBO | 10.2210/pdb5QBO/pdb |
| 5QBP | 10.2210/pdb5QBP/pdb |

| PDB ID | PDB DOI |
|---|---|
| 5QBQ | 10.2210/pdb5QBQ/pdb |
| 5QBR | 10.2210/pdb5QBR/pdb |
| 5QBS | 10.2210/pdb5QBS/pdb |
| 5QBT | 10.2210/pdb5QBT/pdb |
| 5R1T | 10.2210/pdb5R1T/pdb |
| 5R1U | 10.2210/pdb5R1U/pdb |
| 5R1V | 10.2210/pdb5R1V/pdb |
| 5R1W | 10.2210/pdb5R1W/pdb |
| 5R1X | 10.2210/pdb5R1X/pdb |
| 5R1Y | 10.2210/pdb5R1Y/pdb |
| 5R1Z | 10.2210/pdb5R1Z/pdb |
| 5R20 | 10.2210/pdb5R20/pdb |
| 5R21 | 10.2210/pdb5R21/pdb |
| 5R22 | 10.2210/pdb5R22/pdb |
| 5R23 | 10.2210/pdb5R23/pdb |
| 5R24 | 10.2210/pdb5R24/pdb |
| 5R25 | 10.2210/pdb5R25/pdb |
| 5R26 | 10.2210/pdb5R26/pdb |
| 5R27 | 10.2210/pdb5R27/pdb |
| 5R28 | 10.2210/pdb5R28/pdb |
| 5R29 | 10.2210/pdb5R29/pdb |
| 5R2A | 10.2210/pdb5R2A/pdb |
| 5R2B | 10.2210/pdb5R2B/pdb |
| 5R2C | 10.2210/pdb5R2C/pdb |
| 5R2D | 10.2210/pdb5R2D/pdb |
| 5RBO | 10.2210/pdb5RBO/pdb |
| 5RBP | 10.2210/pdb5RBP/pdb |
| 5RBQ | 10.2210/pdb5RBQ/pdb |
| 5RBR | 10.2210/pdb5RBR/pdb |
| 5RBS | 10.2210/pdb5RBS/pdb |
| 5RBT | 10.2210/pdb5RBT/pdb |
| 5RBU | 10.2210/pdb5RBU/pdb |
| 5RBV | 10.2210/pdb5RBV/pdb |
| 5RBW | 10.2210/pdb5RBW/pdb |
| 5RBX | 10.2210/pdb5RBX/pdb |
| 5RBY | 10.2210/pdb5RBY/pdb |
| 5RBZ | 10.2210/pdb5RBZ/pdb |
| 5RC0 | 10.2210/pdb5RC0/pdb |
| 5RC1 | 10.2210/pdb5RC1/pdb |
| 5RC2 | 10.2210/pdb5RC2/pdb |
| 5RC3 | 10.2210/pdb5RC3/pdb |
| 5RC4 | 10.2210/pdb5RC4/pdb |
| 5RC5 | 10.2210/pdb5RC5/pdb |
| 5RC6 | 10.2210/pdb5RC6/pdb |
| 5RC7 | 10.2210/pdb5RC7/pdb |
| 5RC8 | 10.2210/pdb5RC8/pdb |
| 5RC9 | 10.2210/pdb5RC9/pdb |

| PDB ID | PDB DOI |
|---|---|
| 5RCA | 10.2210/pdb5RCA/pdb |
| 5RCB | 10.2210/pdb5RCB/pdb |
| 5RCC | 10.2210/pdb5RCC/pdb |
| 5RCD | 10.2210/pdb5RCD/pdb |
| 5RCE | 10.2210/pdb5RCE/pdb |
| 5RCF | 10.2210/pdb5RCF/pdb |
| 5RCG | 10.2210/pdb5RCG/pdb |
| 5RCI | 10.2210/pdb5RCI/pdb |
| 5SAK | 10.2210/pdb5SAK/pdb |
| 5SAL | 10.2210/pdb5SAL/pdb |
| 5SAM | 10.2210/pdb5SAM/pdb |
| 5SAN | 10.2210/pdb5SAN/pdb |
| 5SAO | 10.2210/pdb5SAO/pdb |
| 5SAP | 10.2210/pdb5SAP/pdb |
| 5SAQ | 10.2210/pdb5SAQ/pdb |
| 5SAS | 10.2210/pdb5SAS/pdb |
| 5SAT | 10.2210/pdb5SAT/pdb |

**Movie S1** EP-TL00150 temperature ramp-up experiment

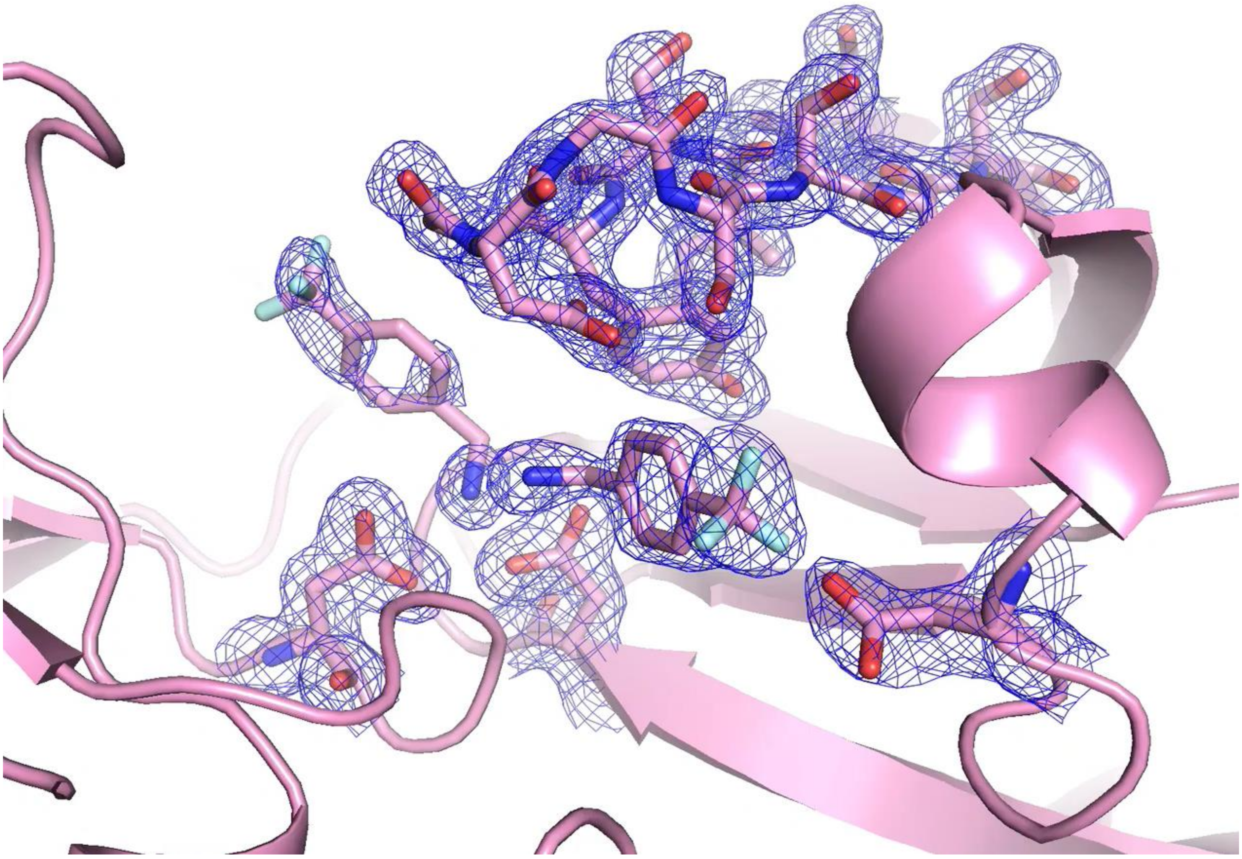

**Movie S2** EP-TL00150 temperature ramp-down experiment

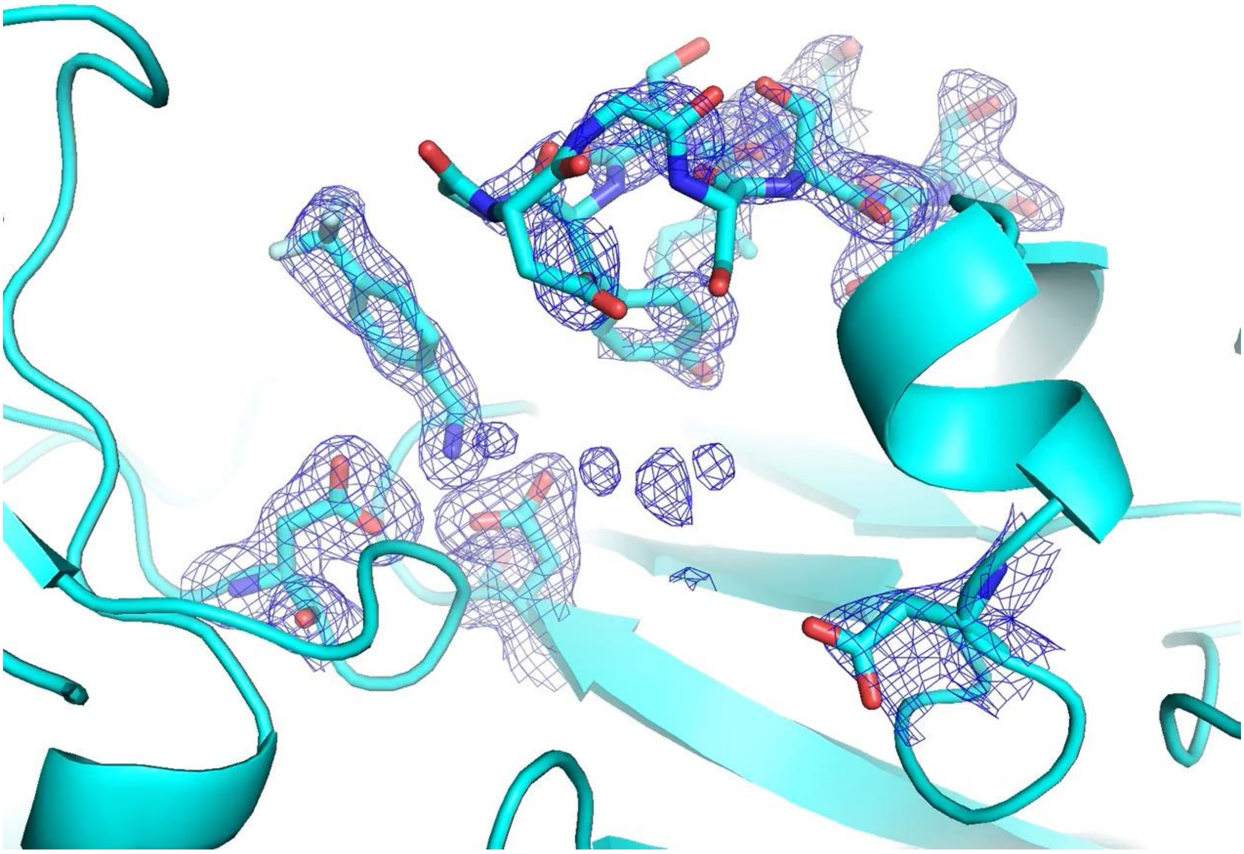

Supplement: Supplementary file 1 [file d-78-00964-sup1.pdf]
